# Neural general circulation models for modeling precipitation

**DOI:** 10.1126/sciadv.adv6891

**Published:** 2026-01-07

**Authors:** Janni Yuval, Ian Langmore, Dmitrii Kochkov, Stephan Hoyer

**Affiliations:** Google Research, Mountain View, CA, USA.

## Abstract

General circulation models (GCMs) struggle to accurately simulate precipitation, particularly extremes and the diurnal cycle, which are crucial for both human activities and natural processes. Although hybrid models combining machine learning and physics offer a promising avenue to improve the simulation of precipitation, they have yet to outperform existing GCMs. Here, we present a hybrid model built on the differentiable NeuralGCM framework. This differentiability facilitates direct training on satellite-based precipitation observations, unlike previous attempts at hybrid models that relied on high-resolution simulations as training data. Our model runs at 2.8° resolution and, in the context of climate, demonstrates substantial improvements over existing GCMs, the ERA5 reanalysis, and a global cloud-resolving model in simulating precipitation. In the context of mid-range precipitation forecasting, it outperforms the ECMWF ensemble. This advance paves the way for more reliable simulations of current climate and demonstrates how training on observations can be used to improve GCMs.

## Teaser

Improving precipitation representation in climate simulations with a hybrid model trained on precipitation observations.

## INTRODUCTION

General circulation models (GCMs) are essential tools for understanding climate change and its impacts, yet they exhibit substantial limitations in accurately representing precipitation, a key variable with profound societal implications. These limitations manifest in both the spatial and temporal dimensions and are especially severe when dealing with extreme precipitation. Spatially, although there has been some modest progress in reducing precipitation biases from CMIP5 to CMIP6 (*1*), biases in historical simulations relative to observations (*2*) can still be as large as the projected changes themselves (*3*) in certain regions, undermining confidence in model projections. Temporally, GCMs struggle to accurately capture the diurnal cycle of precipitation (*4*–*6*), a factor influencing various hydrological processes. The difficulties in accurately simulating precipitation extremes limits our ability to reliably assess regional changes, which are essential for effective climate adaptation planning. Despite the critical societal implications of changes in precipitation (*7*, *8*), there has been little improvement from CMIP5 to CMIP6 in simulating these high-impact events (*9*). This highlights an urgent need to improve the fidelity of precipitation simulations in GCMs.

Precipitation biases in current GCMs are largely attributed to deficiencies in deep convection parameterization schemes (*10*, *11*). To address this, three main approaches have been explored:

1) Kilometer-scale global storm-resolving models (*12*, *13*), although promising, remain computationally prohibitive for long-term climate simulations spanning several decades and still exhibit their own limitations (*14*, *15*).

2) Purely machine learning (ML)–based atmospheric models have shown excellent results for short-term forecasting (*16*, *17*). Recent works have demonstrated the feasibility of running long-term simulations (*18*, *19*) and training models directly on satellite-based precipitation observations (*20*). However, these models have yet to outperform traditional GCMs in terms of long-term precipitation statistics (*21*).

3) Hybrid models incorporating ML parameterizations can be run within a traditional GCM framework (*22*). Thus far, ML parameterizations in atmospheric models have heavily relied on data from high-fidelity simulations, such as convection-resolving models or super-parameterizations, rather than directly incorporating the vast amount of observational data available from satellites, radiosondes, and ground-based instruments. This dependence arises from the difficulty of directly using observational data to derive subgrid-scale tendencies or fluxes, which are the typical training targets for these parameterizations. Although there have been advancements in hybrid models (*23*–*25*), challenges such as instabilities (*26*), climate drift (*27*), and large biases (*28*, *29*) are common and consequently involve online testing of O(100) members within a GCM (*30*). Overall, under realistic conditions, hybrid models are still not competitive with existing GCMs for simulations of climate. Moreover, as long as ML parameterizations depend on high-fidelity simulations rather than observations, they will inevitably inherit the biases present in those simulations.

Recently, a hybrid modeling approach has been combined with differentiable dynamical core to enable end-to-end (i.e., “online”) training. This led to the development of NeuralGCM (*31*), a hybrid model trained on ERA5 (*32*) data. NeuralGCM demonstrated the ability to run decadal simulations (albeit with occasional instabilities), exhibiting lower temperature biases in 40-year runs compared to AMIP-class models, along with a realistic seasonal cycle and state-of-the-art weather prediction skill. However, NeuralGCM trained solely on ERA5 data inherits all of the associated limitations, such as deficiencies in reproducing extreme precipitation events (*33*) and the diurnal cycle of precipitation (*34*).

Building on the NeuralGCM differentiable framework, we develop a hybrid model trained directly on satellite-based precipitation observations. By leveraging observational data, we demonstrate substantial improvements in precipitation simulation both for weather forecasting and on simulations of climate compared to CMIP6 models, ERA5 reanalysis, and a global cloud-resolving model. Furthermore, our model is orders of magnitude faster than traditional GCMs as it simulates ~1200 simulated years/day on a single tensor processing unit (TPU), which facilitates large-ensemble forecasting. This is notably faster than standard physics-based atmospheric models used for climate modeling such as CAM6, which simulates 14 simulated years/day on 1280 CPU cores (*35*).

### Training a hybrid model from observations

**Fig. 1.**
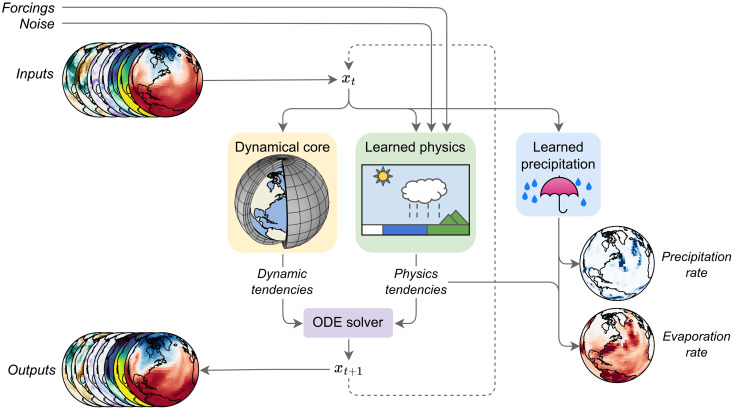
Overall model structure. Inputs are encoded into the model state *x_t_*. This state is fed into the dynamical core and the learned precipitation module. Along with forcings and noise, the state is also used as input to the learned physics module. The dynamical core and learned physics module produce tendencies (rates of change) for an implicit-explicit ordinary differential equation (ODE) solver, which advances the state in time to *x*_*t*+1_. The precipitation module predicts the precipitation rate and, by enforcing water column conservation (Eq. 1), diagnoses the evaporation rate. The updated model state can then be used for the next time step or decoded to produce outputs.

In essence, NeuralGCM comprises two core components (Fig. 1): (i) a differentiable dynamical core and (ii) a learned physics module [i.e., a neural network (NN) parameterization]. This architecture results in a fully differentiable model, facilitating end-to-end (online) training (*31*). Within a differentiable model, optimization of model parameters requires only that the loss can be evaluated on the basis of the ground truth and quantities accessible from the model predictions. This allows learning via minimization of a loss comparing a ground truth, in this case observations, to model an output. Although any observational dataset could theoretically be used, we focus on precipitation as it is a key variable that both models and reanalysis data struggle to simulate accurately.

The process of training NeuralGCM models with satellite-based precipitation observations follows the stochastic training approach of (*31*). All NeuralGCM models presented in this work are trained from scratch. This training minimizes the continuous ranked probability score (CRPS) (*36*) between predicted weather trajectories and the ground truth. We gradually increase the rollout length of these trajectories from 6 hours to 5 days. The trajectories are sampled from ERA5 for atmospheric variables and evaporation and from the Integrated Multi-satellitE Retrievals for Global Precipitation Measurement (IMERG) V07 “final” dataset (*37*) for precipitation. However, to incorporate precipitation optimization while preserving physical consistency and stability, we introduced several key modifications to the NeuralGCM models, as detailed below.

### Atmospheric water budget

The original version of NeuralGCM (*31*) does not explicitly represent precipitation and evaporation. Instead, only the net precipitation minus evaporation (*P* − *E*) is diagnosed using the column water budget (Eq. 1; Materials and Methods). Our objective now is to incorporate a precipitation variable in a manner consistent with the water budget, ensuring plausible values for both evaporation and precipitation. To achieve this, we introduce an NN that predicts precipitation rate from the atmospheric column state (eq. S3) and diagnose evaporation by enforcing the column water budget (eq. S4). In Supplementary Text, we also present an alternative NeuralGCM configuration, referred to as NeuralGCM-evap, which uses an NN to predict evaporation rate from surface variables, with precipitation diagnosed by enforcing the column water budget (eq. S2). We find that NeuralGCM-evap is, in many aspects, superior to the presented model, but one disadvantage is that it does not enforce nonnegative precipitation.

We optimize for temperature, geopotential, zonal and meridional wind, specific humidity, specific water/ice cloud variables, hourly evaporation rate (from ERA5), and 6-hour accumulated precipitation (from IMERG). Optimization occurs every 6 hours, and the model we train has a 2.8° grid spacing.

Simultaneously optimizing NeuralGCM for both IMERG precipitation and ERA5 data presents inherent challenges. This arises from the inconsistency between ERA5 precipitation (and its associated moisture budget) and IMERG precipitation, where ERA5 often exhibits substantial deviations from IMERG, even when both datasets are coarse-grained to a 2.8° resolution (fig. S1; see Materials and Methods for a description of how we coarse-grain IMERG data in time). Consequently, using both ERA5 water variables (i.e., specific humidity, cloud variables, and evaporation rate) and IMERG precipitation for optimization introduces conflicting objectives. In Supplementary Text and in fig. S2, we demonstrate the potential advantages of incorporating physically consistent representations of precipitation and evaporation within NeuralGCM (rather than predicting precipitation without considering the column water budget).

Given our primary goal of enhancing precipitation representation, we have opted to slightly relax the constraint on accurately simulating specific humidity from ERA5 by reducing the corresponding loss weight (see Materials and Methods for how we determine loss weights) while still emphasizing both precipitation and evaporation. This relaxation is supported by the fact that ERA5 specific humidity exhibits nonnegligible differences compared to observations (*38*, *39*), justifying a greater tolerance for deviations from ERA5 in our model. In the Supplementary Text, we also describe several additional modifications to NeuralGCM, which enhance its stability, as well as limitations of our model.

## RESULTS

We train a NeuralGCM model using data from 2001 to 2018. For both weather forecast results and climate results, we regrid all datasets to a 2.8° Gaussian grid using conservative regridding. We then evaluate the skill of the NeuralGCM model for both weather forecasting and long integrations for climate simulations.

We consider both IMERG and the Global Precipitation Climatology Project (*40*) (GPCP; a dataset not used in training) as ground truth for precipitation. These datasets were chosen due to their extensive use and established reliability as benchmarks for precipitation in climate science (*14*, *15*, *32*, *41*), providing robust standards for evaluating the performance of NeuralGCM.

The extensive literature comparing precipitation datasets demonstrates that IMERG and GPCP generally outperform reanalysis data, particularly ERA5, across various metrics and timescales. These include evaluations of diurnal cycles (*42*), extreme precipitation (*43*), and monthly or longer accumulations compared to gauge measurements (*43*–*45*). However, discrepancies exist in assessments of daily or shorter timescales, with some studies favoring IMERG over ERA5 in certain regions (*43*, *46*) whereas others suggest that ERA5 may be more accurate in specific locations (*44*).

It is important to acknowledge that all precipitation products have inherent limitations (*47*). Specifically, IMERG’s calibration process can lead to underestimation of light precipitation and overestimation of heavy precipitation (*48*). However, using coarser spatiotemporal scales, as in this study, generally improves agreement between precipitation products (*49*), particularly between the NOAA Multi-Radar Multi-Sensor system (*50*) and IMERG (*51*) and between IMERG and gauge measurements at subdaily timescales (*52*).

One major limitation of our modeling framework is that obtaining models that are reliably stable over long rollouts requires training ~50 to 100 models. Although not all models were trained with the same configuration (as we identified several stability improvements during the development process; see the Supplementary Materials), it is likely that one would still need to train tens of models to find another model with comparable stability. Furthermore, we focused on the 2.8° resolution as it represents a practical balance between our scientific goal of simulating long climate timescales, the substantial computational resources required for higher resolutions, and the greater challenge of achieving multidecadal stability in such models.

### Medium-range precipitation forecasting

For weather forecasting, we use the WeatherBench2 (*53*) code to evaluate an ensemble of 50 NeuralGCM forecasts for 732 initial conditions at noon and midnight UTC (universal time coordinated) spanning the year 2020, which was held out from the training data. We compare NeuralGCM results to those from the 50-member ECMWF ensemble (ENS) and probabilistic climatology (Materials and Methods) and show example snapshot errors for both models in fig. S3.

**Fig. 2.**
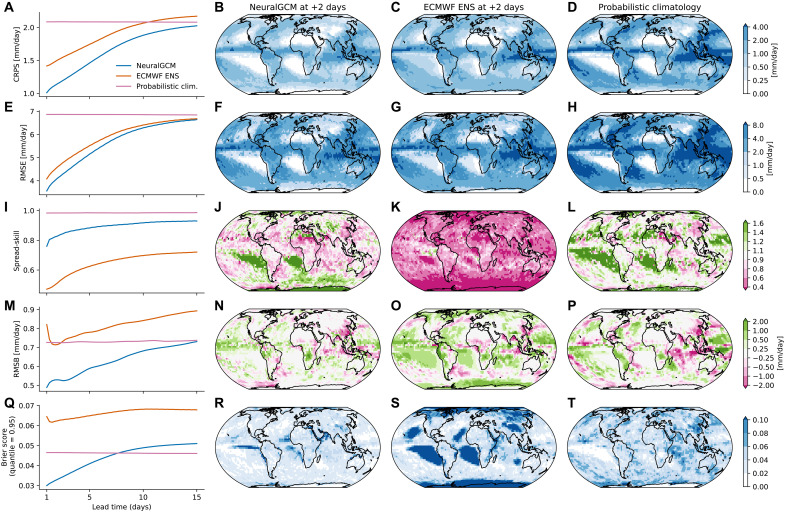
Precipitation forecasting accuracy scores for 24-hour accumulated precipitation, evaluated against IMERG. Area-weighted mean, calculated over all longitudes and latitudes between −60° and 60° for (**A**) CRPS, (**E**) ensemble mean RMSE, (**I**) spread-skill ratio, (**M**) RMSB, and (**Q**) Brier score (0.95 quantile). Comparisons are shown for NeuralGCM, the ECMWF ensemble, and probabilistic climatology (see Materials and Methods). Spatial distributions of (**B** to **D**) CRPS, (**F** to **H**) RMSE, (**J** to **L**) spread-skill ratio, (**N** to **P**) RMSB, and (**R** to **T**) Brier score (0.95 quantile) for NeuralGCM, the ECMWF ensemble, and probabilistic climatology on the second day of forecasting.

We find that NeuralGCM at 2.8° outperforms ENS in precipitation prediction across all 15 forecast days in terms of CRPS, ensemble-mean root-mean-square bias (RMSB), spread-skill ratio, and Brier score (0.95 quantile; see Materials and Methods). These results hold when evaluated against IMERG for 24-hour accumulated precipitation (Fig. 2), including when evaluations are restricted to land regions (fig. S4), and hold for 6-hour accumulated precipitation (fig. S5). NeuralGCM also outperforms ENS when evaluated against 24-hour accumulated precipitation from GPCP (fig. S6). NeuralGCM shows higher skill than probabilistic climatology for CRPS and root-mean-square error (RMSE) for 15 days but has a larger Brier score 7 days. NeuralGCM provides reasonable predictions for other variables but underperforms ENS, as expected given the low resolution of the current NeuralGCM configuration (fig. S7). Sub–6-hour precipitation accumulations in NeuralGCM (but not NeuralGCM-evap) also show unrealistic oscillations in intensity, particularly during the first day of forecasting (fig. S8). In fig. S9, we also compare NeuralGCM to GenCast (*54*). This comparison requires careful interpretation as GenCast was optimized on ERA5 precipitation while NeuralGCM was optimized on IMERG. Therefore, one cannot conclude from this analysis which model has a better forecast or which method (hybrid versus ML-only) is more suitable for precipitation forecasting.

### Precipitation in climate simulations

To test the skill of NeuralGCM in simulating precipitation for climate simulations, we conducted 20-year simulations using 37 initial conditions spaced every 10 days throughout the year 2001. For these simulations, we prescribed historical sea surface temperatures (SSTs) and sea ice concentrations. All 37 initial conditions remained stable for the full 20-year duration for the precipitation model presented in the main text. In addition, we ran a simulation initialized on 30 December 2018, and in the Supplementary Materials, we include figures that repeat the analysis only for years 2019 to 2022, which NeuralGCM was not trained on, and we compare the results from these years to results from a run on years that we did train to see whether the model overfits.

**Fig. 3.**
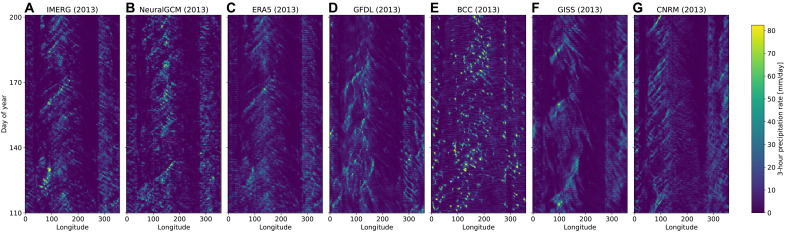
Hovmöller tropical precipitation diagram for different models. Precipitation is averaged between latitudes −5° and 5°. (**A**) IMERG, (**B**) NeuralGCM, (**C**) ERA5, and (**D** to **G**) four CMIP model are shown for historical runs for 91 days starting on 20 April 2013. NeuralGCM run shown was initialized on 27 December 2001. All models were coarse-grained to 2.8° before plotting.

**Fig. 4.**
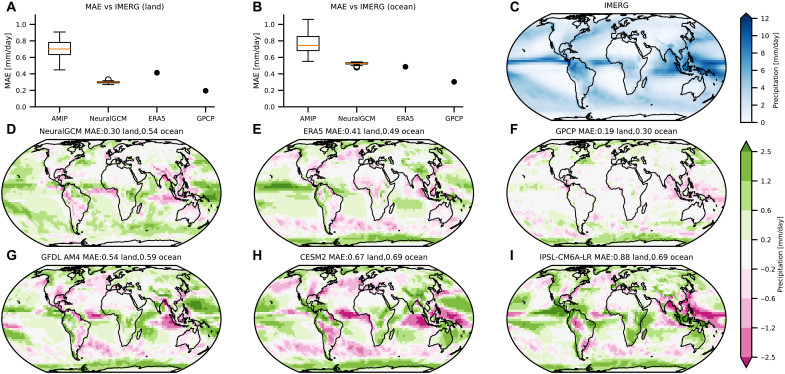
Bias in mean precipitation averaged over 2002 to 2014. (**A** and **B**) Box plots showing the MAE relative to IMERG for 37 NeuralGCM runs (initialized during 2001), 37 CMIP6 AMIP experiments (model details in Materials and Methods), ERA5, and GPCP (*40*) over (A) land and (B) ocean. In the box plots, the red line indicates the median; the box delineates the interquartile range (IQR); whiskers extend to 1.5 × IQR; and outliers are shown as dots. (**C**) IMERG mean precipitation averaged over 2002 to 2014. (**D** to **I**) Bias in mean precipitation from NeuralGCM, ERA5, GPCP, and three CMIP6 AMIP experiments. Global MAE (in mm/day) is shown for land and ocean regions.

**Fig. 5.**
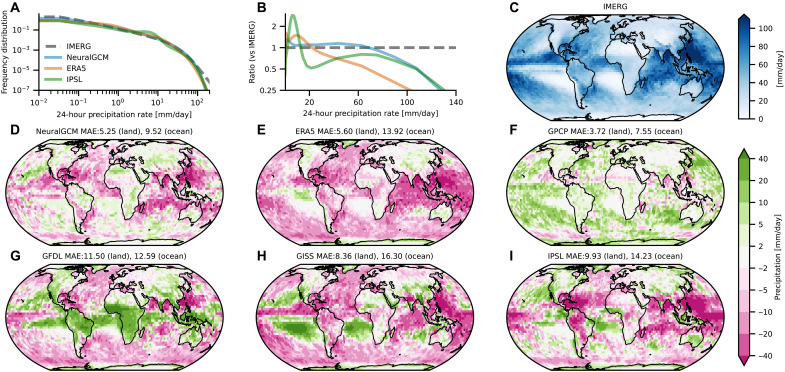
Tropical precipitation rate distribution and annual maximum daily precipitation (Rx1day) averaged over 2002 to 2014. (**A**) Frequency distributions of 24-hourly precipitation rate for IMERG (*37*), NeuralGCM, ERA5, and IPSL-CM6A-LR (historical run) in the tropics (latitudes −20° to 20°). (**B**) Relative distribution normalized by the IMERG value. (**C**) IMERG Rx1day calculated over 2002 to 2014. (**D** to **I**) Bias in Rx1day for NeuralGCM, ERA5, GPCP (*40*), and various CMIP6 historical simulations, relative to IMERG. Global MAE relative to IMERG is shown for land and ocean regions (in mm/day). The NeuralGCM simulation was initialized on 27 December 2001. All models were coarsened to a 2.8° resolution.

**Fig. 6.**
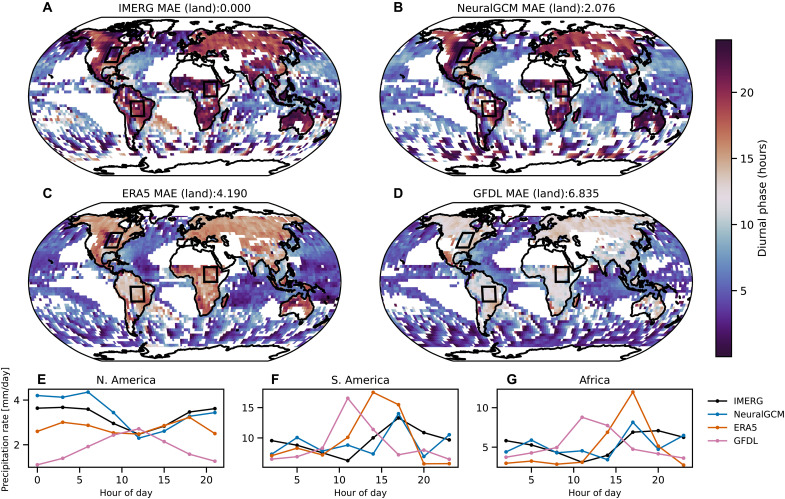
Diurnal cycle of summertime precipitation (2002 to 2014). (**A** to **D**) Local solar time (LST) of maximum precipitation during the summertime (July in the Northern Hemisphere and January in the Southern Hemisphere) derived from the diurnal harmonic for (A) IMERG, (B) NeuralGCM, (C) ERA5 reanalysis, and (D) GFDL AMIP simulation. Regions where either the monthly mean precipitation is less than 0.75 mm/day or the diurnal amplitude ratio (amplitude normalized by mean precipitation) is less than 0.1 are masked in white. MAE is calculated only above land. (**E** to **G**) Summertime diurnal cycle of precipitation (2002 to 2014) over subregions of (E) North America, (F) South America, and (G) Africa (indicated by rectangles in the maps).

We compared various aspects of precipitation in our model to CMIP6 models, ERA5 reanalysis data, and GFDL’s X-SHiELD global cloud-resolving model (*55*). These included Hovmöller diagrams of tropical precipitation (Fig. 3), mean precipitation (Fig. 4), extreme precipitation and precipitation rate (Fig. 5), diurnal cycle (Fig. 6), and the time-space spectrum (fig. S10).

To investigate the sensitivity of extreme precipitation to global mean temperature changes within NeuralGCM, we conducted an extended analysis comprised 732 ensemble runs of 22 years each. All but one of these runs remained stable for the full simulation period. The results of this analysis are presented in the Supplementary Text and illustrated in fig. S36.

Unless stated otherwise, we always use the NeuralGCM simulation initialized on 27 December 2001 for comparison. When comparing against X-SHiELD, we use the available dates in X-SHiELD (18 January 2020 to 17 January 2021) for all relevant models. When comparing against AMIP or historical runs, we compare the years 2002 to 2014 (2014 is the last year that is available for AMIP runs).

To visually demonstrate the differences between models, we show a Hovmöller diagram (*56*) of 3 months of tropical precipitation from IMERG, NeuralGCM, ERA5, and several models from CMIP6 historical runs (Fig. 3) and to X-SHiELD in fig. S13. Qualitatively, NeuralGCM exhibits the most similar structure to IMERG, both in terms of spatial structure and amplitude. All other models show substantial differences in both precipitation magnitude and spatiotemporal structure. ERA5, because of its assimilation process, has a very similar spatiotemporal structure to IMERG but fails to capture heavy precipitation rates. In the following analysis, we quantify further aspects of the simulated precipitation and show that NeuralGCM is not only visually compelling but also statistically superior to the other models.

### Mean precipitation

Figure 4 shows the mean precipitation averaged over 2002 to 2014 for NeuralGCM, ERA5, and 37 CMIP6 AMIP experiments, compared to IMERG observations. Analysis of 37 NeuralGCM runs reveals a global mean absolute error (MAE) of 0.45 mm/day (0.30 mm/day over land and 0.52 mm/day over ocean), compared to 0.74 mm/day (0.76 mm/day over land and 0.70 mm/day over ocean) for 37 AMIP runs, representing a 40% error reduction. Notably, NeuralGCM achieves a similar MAE to ERA5, which is particularly impressive given that NeuralGCM was run freely (forced by SST and sea ice extent), whereas ERA5 assimilated observations every 12 hours. However, we note that NeuralGCM has noteworthy regional biases where ERA5 is more skillful, such as a northward-shifted ITCZ (intertropical convergence zone) over the equatorial Atlantic Ocean and a dipole bias over the equatorial Indo-Pacific region. This superior performance of NeuralGCM compared to AMIP simulations persists across individual seasons (figs. S14 to S17) and when evaluated against GPCP data, which NeuralGCM was not trained on (fig. S18). In fig. S19, we analyze the mean precipitation bias over the 4-year holdout period. The results show that the bias in the holdout years is comparable to the training years.

### Precipitation extremes and precipitation rate distribution

We examine the model’s ability to reproduce the frequency distribution of 24-hourly precipitation rates, a challenging aspect of precipitation simulation that is sensitive to the choice of convection scheme (*10*) and often poorly represented in CMIP-class models (*57*). We estimate frequency distribution using 50 equally spaced bins in the logarithm of the precipitation rate, with the lowest bin starting at 0.03 mm/day and the largest bin at 240 mm/day. We normalize the distribution such that it integrates to one when considering the whole distributions (including rates below 0.03 mm/day). We compare the frequency distributions of NeuralGCM, ERA5, and a single CMIP6 model (IPSL-CM6A-LR) to that of IMERG. We show results for IPSL-CM6A-LR as a representative example of a CMIP6 model to maintain clarity in the figure, but we acknowledge that different models have different distributions.

We find that the NeuralGCM frequency distribution of precipitation rates in the tropics is closer to the distribution from IMERG for both light and extreme precipitation than that of ERA5, IPSL-CM6A-LR (Fig. 5, A and B), and X-SHiELD (fig. S11). However, NeuralGCM underestimates the most extreme precipitation rates, which is partly due to their nature as grid-scale events (see also fig. S1). When the models are further regridded to a 5.6° resolution, NeuralGCM more closely follows the extreme precipitation rate occurrences in IMERG (fig. S12).

To assess the ability of NeuralGCM to simulate the spatial patterns of extreme precipitation, we use the annual maximum daily precipitation at each grid point (often referred to as the Rx1day index; Fig. 5). We find that NeuralGCM represents Rx1day more accurately than ERA5 and the three CMIP6 models included in this comparison, 38 to 55% reduction in MAE over land compared to the CMIP6 models. NeuralGCM’s MAE is only 25% larger than GPCP’s MAE, which, as another observation-based product, provides an estimate of observational uncertainty in IMERG. Furthermore, NeuralGCM outperforms ERA5 and CMIP6 simulations when evaluated for the percent deviation from IMERG Rx1day (fig. S27 highlights regions outside the tropics). We find similar conclusions when studying the 99.9th percentile (fig. S28). We also analyze the bias in extreme precipitation for the 4-year holdout period (fig. S29), defining extremes as the 99th percentile to ensure robust statistics over the shorter window. The bias during the 2019 to 2022 holdout period is slightly larger than in some training periods; however, determining whether this is statistically significant is difficult. For instance, the bias during these years is comparable to that observed in the 2010 to 2013 period.

### Diurnal cycle of precipitation

Following previous studies (*6*, *58*), we characterize the diurnal cycle of precipitation by the local solar time (LST) of maximum precipitation and the amplitude of the diurnal and semidiurnal harmonics (see Materials and Methods). Similar to a previous work (*6*), we focus on the warm season in both hemispheres, where the diurnal cycle is more pronounced.

Figure 6 demonstrates that NeuralGCM more accurately captures the timing of peak diurnal precipitation compared to ERA5 and the GFDL AMIP run, particularly over land. In these land regions, both ERA5 and the GFDL AMIP run exhibit a well-documented “early bias,” with precipitation peaking incorrectly around noon (*7*) (for ERA5, however, the bias is smaller). In contrast, NeuralGCM shifts the peak to the late afternoon and evening, in much better agreement with observations. Over ocean areas, all models are in general agreement with observations, capturing the characteristic nighttime or morning maximum in precipitation. NeuralGCM also exhibits a lower overall MAE for the diurnal and semidiurnal amplitude, as well as for the semidiurnal phase (figs. S20 to S22). Over the ocean, however, the diurnal amplitude is captured well by all models, with ERA5 showing a slightly smaller MAE than NeuralGCM (fig. S20). However, as noted previously, the diurnal cycle in NeuralGCM exhibits unrealistic features, with certain times of day experiencing more precipitation than others (Fig. 6, E to G, and fig. S8), likely due to the model being optimized for 6-hourly precipitation accumulation. These unrealistic diurnal features are not present in NeuralGCM-evap (figs. S8 and S33). We also compare the diurnal cycle with the X-SHiELD cloud-resolving model (fig. S23) for 2020, a year on which NeuralGCM was not trained.

## DISCUSSION

By harnessing a differentiable dynamical core and an NN parameterization, NeuralGCM can be trained jointly on ERA5 and observational products, providing a compelling example of how observational knowledge can enhance the fidelity of atmospheric simulations. When trained on satellite-based precipitation from IMERG, NeuralGCM remains stable for decadal simulations and substantially surpasses traditional GCMs and ERA5 in accurately simulating key aspects of precipitation, including its mean state, extremes, and the diurnal cycle.

Although this study used an NN to parameterize all processes unresolved by the dynamical core, future work could explore coupling our differentiable dynamical core with a traditional parameterization suite and optimizing its free parameters. This approach offers the potential to further refine existing parameterizations by leveraging observational data. Moreover, it could reveal inherent limitations in the structure of current parameterizations, guiding the development of more accurate and physically consistent representations of unresolved processes.

Although our model has a lower resolution than typical models used for weather forecasts of precipitation, which limits its immediate practical applications, it demonstrates that a low-resolution hybrid model can substantially outperform ECMWF’s ensemble prediction system in precipitation prediction. In particular, our analysis shows the model’s spread calibration is improved globally compared to the ECMWF ensemble (Fig. 2I). However, the regional variations in the spread-skill ratio also underscore that calibrating the spread perfectly at a local level is a nontrivial task and, as with traditional ensembles, requires further work. This suggests that further improvements in resolution, achieved through statistical downscaling or a higher-resolution model, could yield substantial gains compared to ECMWF’s model.

Our work retains some noteworthy limitations. Although the presented NeuralGCM is much more stable than prior models (*31*), the stable model was still obtained by training multiple models with varying random seeds and choosing the most stable one. Further research is needed to understand and address the factors that influence model stability. Last, developing effective strategies for learning from potentially conflicting datasets is crucial. In this study, we encountered inconsistencies between ERA5 and IMERG, necessitating careful tuning of the loss function. Ideally, future research will also prioritize the development of unified datasets to provide a single, consistent ground truth for model training, thereby avoiding the need for ad hoc adjustments.

## MATERIALS AND METHODS

### Neural networks

#### NN for predicting tendencies

NeuralGCM’s NN parameterization for predicting tendencies adopts the single-column approach common in GCMs, where information from a single atmospheric column is used to predict the impact of unresolved processes within that column. A fully connected NN with residual connections is used for this prediction, with the network weights shared across all columns.

A full description of the NN parameterization (i.e., the NN that predicts tendencies), its architecture, features, and parameters is detailed in the supplementary material of (*31*). The main difference in this work compared to our previous paper is that the parameterization also predicts tendencies for log surface pressure, which improved stability in multiyear simulations.

#### NN for predicting precipitation

Here, we use an additional single-column network to predict precipitation (at 1-hour intervals) but with different parameters and inputs. Overall, the precipitation network is similar to the parameterization network, but it is much smaller. The features and architecture of the precipitation NN are described below.

The core input features to the NN include the vertical profiles of zonal and meridional wind, temperature anomalies, specific humidity, specific cloud ice water content, and specific cloud liquid water content. Unlike in the NN parameterization for predicting tendencies, we do not include the spatial derivatives of these fields as inputs. We also include orography (along with its spatial gradients), a land-sea mask, and an eight-dimensional location-specific embedding vector for each horizontal grid point. This embedding vector aims to represent static, location-specific information related to precipitation (e.g., subgrid orography). It is initialized with random values and optimized during training.

In addition, we use a surface embedding network that receives surface-related inputs, specifically SST and sea ice concentration. Over land and ice where SST is not available, we include the lowest model level temperature and specific humidity. [Full details are provided in (*31*).]

It is important to note that the learned embedding vector and the surface embedding network for the precipitation NN have different parameters than those used in the NN parameterization that predicts tendencies. All features are normalized to have an approximate zero mean and unit variance to improve training dynamics, as described in (*31*).

Similar to the NN parameterization for predicting tendencies, we use a fully connected NN with residual connections (*31*). However, this network predicts only precipitation. We use an Encode-Process-Decode (EPD) architecture (*59*) with three fully connected MLP blocks in the “Process” component (compared to five blocks in the NN parameterization for predicting tendencies).

All input features for the NN that predicts precipitation are concatenated and passed to the “Encode” layer, a linear layer that maps the input features to a latent vector of size 64 (compared to 384 in the NN parameterization for predicting tendencies). Each “Process” block uses a three-layer MLP with 64 hidden units (compared to 384 for the NN parameterization for predicting tendencies) to update the latent vector. Last, a linear “Decode” layer maps the latent vector of size 64 (384 in the NN parameterization for predicting tendencies) to the hourly precipitation rate. A rectified linear unit (ReLU) activation function is then applied to ensure nonnegativity of the predicted precipitation. Including the location-specific embedding vector, the overall number of parameters optimized for the precipitation network is 131,432.

### Variable rescaling for losses

To balance the contributions of different variables to the loss function, we rescaled the losses following a similar approach to that in our previous work (*31*). Specifically, we divided each atmospheric variable by the SD of its temporal difference over 24 hours and applied a time-dependent rescaling function (*31*). However, we reduced the scaling factor for specific humidity by a factor of 100 to discourage the model from closely following ERA5 estimates of specific humidity. This adjustment allowed us to achieve precipitation values closer to IMERG. The scaling factors for precipitation and evaporation were determined empirically to ensure that these variables contributed ~10 and 20%, respectively, to the total loss, whereas specific humidity contributed only 3%.

### Water budget in the NeuralGCM model

Precipitation minus evaporation is diagnosed by integrating the moisture budget tendencies from the NN parameterization for tendencies

$$P-E=\frac{1}{g}\int_{0}^{1}\sum_{i}{\left(\frac{\mathrm{dq}}{\mathrm{dt}}\right)}_{i}^{{\text{NN}}_{\text{tend}}}p_{s}\mathrm{d\sigma}$$ (1)

where σ is the vertical coordinate (which is pressure divided by surface pressure), *p*_s_ is the surface pressure, and $\sum_{i}{\left(\frac{\mathrm{dq}}{\mathrm{dt}}\right)}_{i}^{{\text{NN}}_{\text{tend}}}$ is the sum of the water species (i.e., specific humidity *q*, specific cloud ice $q_{c_{i}}$, and specific liquid cloud water content $q_{c_{l}}$) tendencies predicted by the NN.

### Diurnal cycle of precipitation

Following previous studies (*6*, *58*), we apply Fourier analysis to the diurnal time series of precipitation. (The data are first grouped by hour and averaged.) The 3-hourly precipitation time series, *P*(*t*), t∈{0…,23}, is then represented as

$$P\left(t\right)=S_{0}+S_{1}\left(t\right)+S_{2}\left(t\right)+\text{residual}$$ (2)

and

$$S_{n}=A_{n}\text{sin}\left(\text{nt}+\sigma_{n}\right)$$ (3)

Here, *S*_1_ represents the diurnal cycle, *S*_2_ represents the semidiurnal cycle, *S*_0_ represents the mean precipitation, *A_n_* represents the harmonic amplitude, σ*_n_* represents the phase, and *t* is the LST expressed in radians (i.e., $t=2\pi t_{1}/24$, where *t*_1_ is the LST in hours).

### CMIP6 AMIP and historical runs

The CMIP6 (*60*) data used in this study were obtained from Google’s Public Dataset program stored on Google Cloud Storage.

#### AMIP runs

For the analysis of monthly mean precipitation, we used the following AMIP models (all with member ID r1i1p1f1): GFDL-ESM4, GFDL-CM4, GFDL-AM4, GISS-E2-1-G, IPSL-CM6A-LR, MIROC6, BCC-CSM2-MR, BCC-ESM1, MRI-ESM2-0, CESM2, SAM0-UNICON, CESM2-WACCM, FGOALS-f3-L, CanESM5, INM-CM4-8, EC-Earth3-Veg, INM-CM5-0, MPI-ESM-1-2-HAM, NESM3, CAMS-CSM1-0, MPI-ESM1-2-HR, EC-Earth3, KACE-1-0-G, MPI-ESM1-2-LR, NorESM2-LM, E3SM-1-0, NorCPM1, FGOALS-g3, ACCESS-ESM1-5, TaiESM1, FIO-ESM-2-0, CAS-ESM2-0, CESM2-FV2, CESM2-WACCM-FV2, CMCC-CM2-SR5, EC-Earth3-AerChem, and IITM-ESM. CIESM was excluded from the analysis due to large biases.

For 3-hourly precipitation in Fig. 6 and figs. S10, S20, S21, and S22, we used the GFDL-CM4 (r1i1p1f1) AMIP run.

For the analysis of global mean temperature in figs. S24 and S35, we used the same 22 AMIP models as in (*31*). Specifically, we used the following 17 models with the member ID r1i1p1f1: BCC-CSM2-MR, CAMS-CSM1-0, CESM2, CESM2-WACCM, CanESM5, EC-Earth3, EC-Earth3-Veg, FGOALS-f3-L, GFDL-AM4, GFDL-CM4, GFDL-ESM4, GISS-E2-1-G, IPSL-CM6A-LR, MIROC6, MRI-ESM2-0, NESM3, and SAM0-UNICON. For the remaining five models, we used alternative member IDs: r1i1p1f2 for CNRM-CM6-1 and CNRM-ESM2-1, r2i1p1f3 for HadGEM3-GC31-LL, r1i1p1f3 for HadGEM3-GC31-MM, and r1i1p1f2 for UKESM1-0-LL.

#### Historical runs

Because of the limited availability of 3-hourly or daily precipitation data for AMIP models in Google’s Public Dataset program, we used historical simulations for analyses requiring these temporal resolutions. In Figs. 3 and 5, we used GFDL-CM4, IPSL-CM6A-LR, BCC-CSM2-MR, MRI-ESM2-0, and GFDL-ESM4 (with member ID r1i1p1f1), as well as CNRM-CM6-1, GISS-E2-1-G, and CNRM-ESM2-1 (with member ID r1i1p1f2).

### Comparison with observation-based data

To evaluate the representation of precipitation in simulations, we primarily used the IMERG V07 “final” dataset (*37*), which provides precipitation estimates at a 0.1° spatial resolution and 30-min temporal resolution for the period 2001 to 2023. This dataset uses data from the Global Precipitation Measurement satellite constellation and other data, including monthly surface precipitation gauge analyses. To obtain a spatial resolution comparable to that of NeuralGCM, the data were conservatively regridded from the original 0.1° resolution to a 2.8° grid and averaged over time to provide 3-hourly, 6-hourly, and daily precipitation rates.

IMERG provides instantaneous estimates of precipitation (rather than cumulative values) every 30 min. We converted these to accumulated quantities, taking into account the IMERG documentation’s suggestion: “It is usually best to assume that this rate applies for the entire half-hour period” (https://gpm.nasa.gov/resources/faq/how-intensity-precipitation-distributed-within-given-data-value-imerg). However, IMERG provides these instantaneous values at some point within the 30-min interval after the timestamp. When time-aggregating the data, we assumed that the *Y*-minute accumulation rate at time *X* is calculated by taking the IMERG values at times [*X*-*Y*+30 min, *X*-*Y*+60 min, ..., *X*]. This calculation potentially shifts the accumulation by up to 30 min. This shift could slightly affect the weather evaluation scores but should not have a large impact on the climate-related plots. To verify the robustness of our weather evaluations, we also evaluated our ensemble weather forecast against the GPCP dataset (fig. S6) and found similar results to those obtained using IMERG.

Our analysis also incorporates the GPCP (*40*) One-Degree Daily dataset, which provides precipitation estimates by merging data from multiple satellite sources, and surface rain gauge measurements. Over land, these satellite-based estimates are further refined using monthly rain gauge measurements. We also conservatively regrid this dataset to a 2.8° grid.

We emphasize that IMERG and ERA5 are derived from fundamentally different methodologies. ERA5 is a reanalysis product where precipitation is an output of a numerical weather model that assimilates observations. IMERG, conversely, is a satellite-based retrieval product built more directly from observations. Given these distinct production pipelines, it is natural that their precipitation fields differ, particularly for instantaneous snapshots (e.g., 3-hourly) as shown in fig. S1.

### Brier scores

We compute Brier scores comparing the (50 member) ensemble tail probabilities with observational datasets. To do this, we first compute thresholds λ*_i_*, corresponding to quantiles $q_{i}=\left(0.95,0.99\right)$ (separately for every latitude/longitude/dayofyear). In other words, with *Y* ground truth, the historical $P\left[Y<\lambda_{i}\right]=q_{i}$. The Brier score at each latitude/longitude lead time ℓ is then defined with an average over initial times T as

$$\frac{1}{|T|}\sum_{t\in T}{\left|\frac{1}{50}\sum_{n=1}^{50}1_{X_{t+\ell }^{\left(n\right)}>\lambda_{i}}-1_{Y_{t+\ell }>\lambda_{i}}\right|}^{2}$$

Above, $1_{X_{t+\ell }>\lambda_{i}}=1$ when $X_{t+\ell }>\lambda_{i}$ and =0 when $X_{t+\ell }\leq \lambda_{i}$, $\left\{X_{t+\ell }^{\left(1\right)},\ldots X_{t+\ell }^{\left(50\right)}\right\}$ is the 50-member ensemble forecast value at the latitude/longitude and lead time ℓ, and $Y_{t+\ell }$ is the corresponding ground truth.

### Probabilistic climatological forecasts

As an additional baseline, we generate a size 50 ensemble of forecasts *X_clim_* by sampling historical IMERG data *X_hist_*. Creation of the forecast at initial time *t* starts by choosing a random source initial time *s*. The forecast at lead time τ is then $X_{\text{clim}}\left(t+\tau \right)=X_{\text{hist}}\left(s+\tau \right)$. To choose the initial time *s*, we first choose s.year uniformly in 1990 to 2019 (for ERA5) and 2001 to 2019 (for IMERG). Second, we choose s.dayofyear uniformly in [t.dayofyear − 7, t.dayofyear + 7]. Time of day is unchanged, and sampling is done without replacement.

## Supplementary Materials

### This PDF file includes:

Supplementary Text Figs. S1 to S36 References
