# Supplementary material for: Neural general circulation models for modeling precipitation
Source: Sci Adv. 2026 Jan 7;12(2):eadv6891. doi: 10.1126/sciadv.adv6891 (PMC12778041; doi:10.1126/sciadv.adv6891)
Supplement: Supplementary file 1 — Supplementary Text Figs. S1 to S36 References [file sciadv.adv6891_sm.pdf]

Science Advances
AAAS

Supplementary Materials for

Neural general circulation models for modeling precipitation

Janni Yuval *et al.*

Corresponding author: Janni Yuval, janniyuval@google.com

Sci. Adv. **12**, eadv6891 (2026)
DOI: 10.1126/sciadv.adv6891

This PDF file includes:

Supplementary Text
Figs. S1 to S36
References

Supplementary Text

Limitations of the current NeuralGCM model

There are several notable caveats in the current version of NeuralGCM which we highlight here:

Temperature bias and spread of simulations. The current version of NeuralGCM exhibits a global mean temperature bias of approximately 0.5K at 850hPa with respect to ERA5 (fig. S24). While substantial, this bias is smaller than that in some AMIP models. Another key issue with NeuralGCM precipitation model, also present to some extent in the previous version, is the large spread in global mean temperature within the NeuralGCM ensemble. This spread is substantially larger than that obtained in physics-based models, such as MIROC6 (fig. S24). Interestingly, the NeuralGCM-evap, which was trained to predict evaporation and diagnose precipitation, not only has a substantially smaller ensemble spread but also exhibits a reduced global mean temperature bias this spread is substantially smaller (fig. S35).

Unrealistic instantaneous evaporation The NeuralGCM presented here was trained to optimize both 6-hourly evaporation rates and accumulated precipitation. For the model described in the main text, where precipitation is predicted and evaporation diagnosed, the annual mean diurnal cycle of evaporation is consistent with that of ERA5 (fig. S25). However, unrealistic artifacts are evident in instantaneous snapshots of evaporation (fig. S26). This likely arises from the indirect estimation of evaporation, which hinders the model's ability to achieve smooth evaporation fields. Notably, these artifacts are absent in the NeuralGCM-evap (configuration where evaporation is predicted and precipitation diagnosed; fig. S26). As discussed earlier, NeuralGCM is trained on potentially conflicting datasets. This may further contribute to the challenges in obtaining consistent values for both evaporation and precipitation simultaneously.

Unrealistic precipitation at frequencies higher than 6 hours As was discussed in the manuscript and was shown in Figs. 6, S8 , the NeuralGCM model that is introduced in the manuscript exhibits unrealistic diurnal features, with certain times of day experiencing considerably more precipitation than others (this is especially noticeable in the first day). Therefore, we recommend against using this configuration at frequencies higher than 6-hourly. These issues, however, do not occur in NeuralGCM-evap (Figs. S8,S33).

Stability overall dependent on the initial seed. We introduced a NeuralGCM (and NeuralGCM-

evap) model capable of stable long-term simulations. These models were stable for all 37 initial conditions tested over a 20-year period, and in a larger experiment, 731 out of 732 simulations ran for 22 years without instability. However, during development, we observed that models trained with different random seeds (resulting in different initial model parameters or weights) exhibited notable variations in stability. Specifically, models trained with similar settings but initialized with different seeds demonstrated markedly different stability durations, despite their similar training. We note that improving stability of hybrid models remains a challenging task.

Key changes in NeuralGCM to improve stability

We observed that some instabilities in the previous version of NeuralGCM were related to a drift in the global mean log surface pressure. We found that a similar drift also occurs when running NeuralGCM dynamical core alone with realistic orography (and without the ML parameterization). Furthermore, we found that NeuralGCM models exhibit some sensitivity to initial conditions, even in long integrations. For example, models initialized at different dates could produce slightly different mean temperatures. To mitigate these issues, we allowed the neural network parameterization to modify the log surface pressure prognostic variable and constrained the global mean log surface pressure to remain constant during long integrations. Additionally, we removed the stochastic component of the encoder (excluded the random field input), which further improved stability.

Other changes in NeuralGCM precipitation model

Besides the configuration changes mentioned above, there are two minor differences between the NeuralGCM precipitation model presented here and the one described in (*31*):

- We use a dynamical core time step of 20 minutes (compared to 12 minutes in the 2.8° model presented in (*31*)).
- We include cloud fields as inputs to the decoder, which were mistakenly omitted in the previous version.

Computational resources

To train the specific models we use it took us a bit under two days to train each model using 32 TPUs v5e. During inference we could run $\sim 438,000$ simulation days in 24 hours on a single TPU.

Dispersion relation

fig. S10 presents the dispersion relationships in the zonal wavenumber-frequency domain, computed following the methodology of Wheeler and Kiladis (*61*) using the wavenumber-frequency Python package (*62*). Our analysis is computed using the precipitation field, in contrast to the original study by Wheeler and Kiladis, which used outgoing longwave radiation. Both the symmetric and anti-symmetric components of the Wheeler-Kiladis diagram for NeuralGCM show a close resemblance to the observed dispersion relations, capturing key tropical wave modes such as the Madden-Julian Oscillation (MJO), Kelvin waves, and equatorial Rossby waves. This representation aligns more closely with IMERG data than the GFDL model, although NeuralGCM exhibits an unrealistic peak at low frequencies. Notably, the dispersion relation derived from ERA5 demonstrates an even closer correspondence to IMERG than NeuralGCM.

Results for NeuralGCM evaporation model

We also experimented with a model that uses a neural network to predict evaporation:

$$E = \mathrm{NN}_{\mathrm{evap}}(X), \tag{S1}$$

where $\mathrm{NN}_{\mathrm{evap}}$ is a neural network that predicts evaporation (E), and X represents the inputs to the network. Precipitation (P) is then diagnosed by enforcing water conservation in the column (Eq. 1):

$$P = \frac{1}{g}\int_0^1 \sum_{\mathrm{i}} \left(\frac{dq}{dt}\right)_{\mathrm{i}}^{\mathrm{NN}_{\mathrm{tend}}} p_s d\sigma + \mathrm{NN}_{\mathrm{evap}}(X). \tag{S2}$$

We refer to this model as NeuralGCM-evap. This formulation has the advantage of being more aligned with how current atmospheric models are constructed, where evaporation is calculated using a surface scheme. Furthermore, the neural network employed in this approach is smaller, as it only takes near-surface atmospheric values as input (see below).

One disadvantage of this model is that it can produce negative precipitation values. While we attempted to address this issue, techniques that ensured non-negative precipitation (while predicting evaporation) led to less stable models.

Results for evaporation model

We evaluated NeuralGCM-evap by following the procedure described in the main text, where we conducted 20-year simulations for 37 different initial conditions. Of these, 36 out of 37 initial conditions remained stable and exhibited no drift in global mean temperature (fig. S35).

Overall, the results from this model are also quite compelling, with the major caveat that it produces negative precipitation values (see the precipitation rate frequency distribution in fig. S30). In terms of mean precipitation (fig. S31), extreme precipitation (fig. S32) and diurnal cycle (fig. S33) this model exhibits even better performance than the model presented in the main text. However, this assessment includes negative precipitation values in the calculation of MAE. When these negative values are set to zero, the MAE for mean precipitation increases (fig. S31c). NeuralGCM-evap also exhibits a realistic precipitation also at frequencies higher than 6 hours which it didn't directly train on (Figs. S8, S33) and does not exhibit the unrealistic features present in NeuralGCM at these higher frequencies. Furthermore, NeuralGCM-evap produces a time-space spectrum similar to that of IMERG (fig. S34).

Input features for evaporation neural network

The primary input features to the neural network that predicts evaporation include the surface values of zonal and meridional wind, temperature anomalies, and specific humidity.

Additionally, we incorporate an 8-dimensional location-specific embedding vector for each horizontal grid point. This vector, initialized with random values, is trained to represent unique geographical features. We also utilize a surface embedding network that receives surface-related inputs, specifically sea surface temperature (SST) and sea ice concentration. Over land and ice where SST data are unavailable, the lowest model level temperature and specific humidity are included as input. (Full details are provided in (*31*) which uses a similar surface embedding network.)

Evaporation network architecture

The evaporation network, like the precipitation network, employs an Encode-Process-Decode (EPD) architecture but with a smaller size than the precipitation NN. The "Encode" layer maps the input features to a latent vector of size 8 (compared to 64 for the precipitation network). Each "Process"

block utilizes a 3-layer MLP with 8 hidden units (compared to 64 for the precipitation network). Finally, the "Decode" layer maps the latent vector of size 8 (64 for the precipitation network) to the hourly evaporation rate.

Potential benefits to physical consistency

To illustrate the potential benefits of enforcing consistency with the water budget, we compare the precipitable water (PW) distribution of our model to that of a different NeuralGCM model trained without this constraint. As baselines, we use ERA5, which our models used as a target, and AQUA AIRS measurements (*63*), which were not used in training (AQUA AIRS data was downloaded from `https://disc.gsfc.nasa.gov/datasets/SNDRAQIL3CDCCP_2/summary?keywords=climcaps`).

The unconstrained approach, akin to typical machine learning models, adds a new output (precipitation) without direct interaction with other variables, except through the optimization process. Namely,

$$P = \mathrm{NN}_{\mathrm{precip}}(X) \tag{S3}$$

where $\mathrm{NN}_{\mathrm{precip}}$ is a neural network that predicts precipitation (P), and X represents the inputs to the network (Methods). As expected, the unconstrained model produces a PW distribution similar to that of ERA5, which was used as the training target (Fig. S2).

In contrast, for the model presented in the main manuscript, we also diagnose evaporation (E) by enforcing water conservation in the column (Eq. 1):

$$E = \mathrm{NN}_{\mathrm{precip}}(X) - \frac{1}{g}\int_0^1 \sum_{\mathrm{i}} \left(\frac{dq}{dt}\right)_{\mathrm{i}}^{\mathrm{NN_{tend}}} p_{\mathrm{s}} d\sigma. \tag{S4}$$

Enforcing the water budget constraint results in a feedback into the neural network for tendency prediction. This shifts the PW distribution in the NeuralGCM model towards higher values, which are observed in AQUA AIRS but are not observed in ERA5, albeit with some overestimation at the high end (Fig. S2). This comparison highlights the potential of incorporating physical constraints into machine learning models, as such constraints can influence model dynamics and lead to improved realism.

Extreme precipitation sensitivity to changes in global mean temperature

An outstanding challenge in climate science is quantifying how the intensity of extreme precipitation responds to changes in global mean temperature. This sensitivity is a critical metric for assessing future climate risks, but robustly estimating it from observations on regional scales is difficult because the signal is many times obscured by the noise of natural climate variability. Detecting statistically significant changes in extreme precipitation over relatively short periods necessitates numerous ensemble members, making computational efficiency crucial. The NeuralGCM model described here can simulate approximately 1200 years in 24 hours on a single TPU v4. This enables the feasible execution of O(1000) 20-year realizations using 16 TPU v4 days. (In practice, we use several TPUs in parallel and do not need to wait 16 days for the results.) As a proof of concept on how large ensembles can be used, we use NeuralGCM estimate the sensitivity of annual maximum daily precipitation (Rx1day) to global mean temperature changes. We ran 732 ensemble members for 22 years (two ensemble members initialized every 24 hours during 2001; 731 out of 732 remained stable for the full 22 years of simulations). We use this ensemble to estimate the sensitivity of annual maximum precipitation (Rx1day) to changes in global mean temperature.

We acknowledge that using a specific 20-year period raises questions about the influence of strong internal variability present in the prescribed SSTs. Furthermore, because our analysis relies on prescribed SSTs that contain signals from both natural variability and anthropogenic forcing, it cannot isolate the influence of climate change on extreme precipitation events. Future work will need to incorporate an ocean model to facilitate a more controlled analysis, enabling comparisons of different years with varying SST realizations.

Previous studies have used both models (*64, 65*) and observations (*66, 67*) to estimate the sensitivity of extreme precipitation to changes in global mean temperature. Although there are still large uncertainties regarding the response of extreme precipitation to global mean temperature increase on regional scales, there is ample evidence from both observations and models that, on large spatial scales, there is an overall increase in extreme precipitation globally.

For each grid box, the Rx1day values from all years and ensemble members are regressed against the 850 hPa temperature anomalies (calculated separately for each year) using the Theil-Sen estimator. The regression coefficient is then divided by the mean of the annual-maximum

daily precipitation rate (averaged over the whole ensemble) at that grid box to yield a sensitivity expressed in units of % K^{-1} (Fig. S36). This methodology is similar to that used previously (*67*), with the difference that we use the 850 hPa temperature instead of the near-surface temperature (since NeuralGCM does not output near surface temperature).

Overall, the results shown in fig. S36 are mostly consistent with those obtained from the ensemble mean of CMIP5 models (*64,65*), where there is an overall increase in extreme precipitation in most regions (particularly near the equator in the Pacific). However, there are several regions exhibiting a decrease in Rx1day with increasing global mean temperature, including subtropical regions in the North Atlantic and South Pacific near South America, as well as North Africa and western Australia. There are also some clear differences; for example, NeuralGCM indicates a decrease in the Arabian Peninsula and the Arabian Sea, which is not seen in the ensemble mean of CMIP5 models.

We emphasize that the differences between NeuralGCM and the ensemble mean of CMIP5 models are not necessarily a negative indication, since the true sensitivity is unknown and because an ensemble mean of models attenuates extreme values that occur in individual models.

To make a rough comparison between NeuralGCM and observations over land, we calculate the median sensitivity for each latitudinal band. Similar to observations, we find that over land, all latitudes show an increase in Rx1day with increasing temperature (fig. S36c). Confidence intervals are estimated using 1000 smoothed bootstrap (*68*) iterations at each latitude. First, locations are sampled with replacement. Second, for each location, we randomly draw a slope value from a normal distribution centered at the location's mean slope, and standard deviation obtained from the Theil-Sen regression. The normal sampling step is a smoothing (*68*), which improves the estimate in light of the limited number of points at each latitude. The global mean sensitivity is 4.2% K^{-1} (with 95% confidence intervals calculated from the Theil-Sen regression of 3.9–4.5% K^{-1}), which is slightly lower than the range obtained from some previous work, estimated to be 5–10% K^{-1} (*69, 67*) (with some sensitivity to the exact method of estimation), but still within a reasonable range. We note that observations have a very non-uniform distribution over land, so the comparison is not exactly apples-to-apples. Furthermore, previous work indicates that extreme precipitation responses to global mean temperature are more modest at coarser resolutions (*70*) and lower percentiles (*71*). It is possible that at higher resolutions or higher percentiles, the response

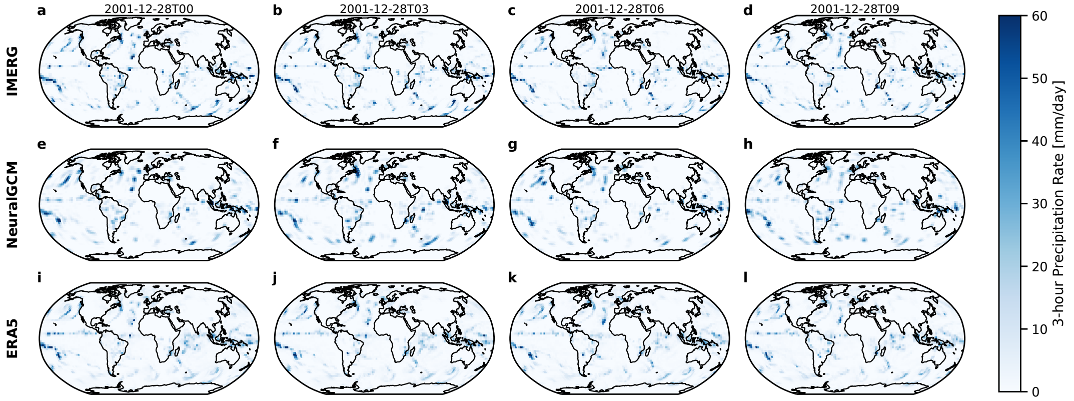

Figure S1: 3-Hourly precipitation rate from IMERG, NeuralGCM and ERA5. NeuralGCM was initialized on 12-27-2001 (24 hours before the first snapshot is shown).

would be more pronounced. To facilitate a reasonable comparison to observations, we mask out regions with Rx1day < 20 mm/day, which effectively masks out North Africa (fig. S36b), where the observational record is practically non-existent.

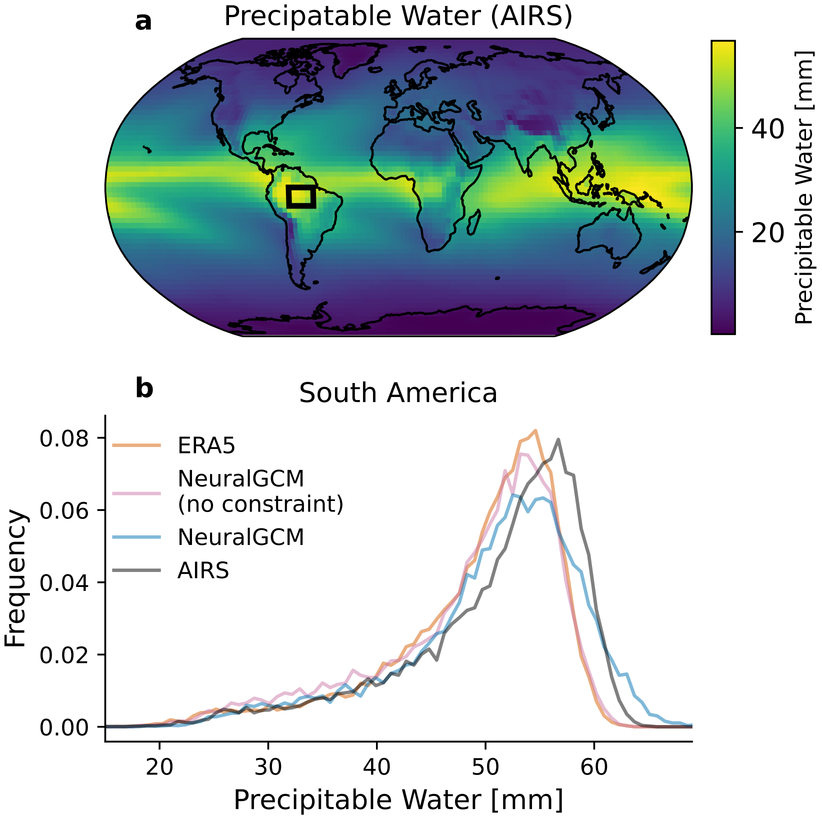

Figure S2: Comparison of precipitable water (PW) from different datasets and models. (a) Time-averaged PW (excluding cloud water) from AQUA AIRS (*63*) measurements at 1:30 PM local time, averaged over 2018–2019. (b) Frequency distribution of PW over South America (latitudes $-10°$ to 0◦, longitudes 292.5° to 307.5°) for ERA5, AQUA AIRS, and two NeuralGCM configurations: (1) "no constraint" – trained to predict precipitation without any constraint on evaporation or the water budget; (2) NeuralGCM model discussed in the manuscript which uses water budget constraint, with optimization of both precipitation and evaporation. AIRS data are from 1:30 PM local time. For ERA5 and NeuralGCM, data are from 19:00 UTC, which roughly corresponds to 1:30 PM local time in the chosen region. NeuralGCM simulations were initialized on January 1, 2018, and run for two years.

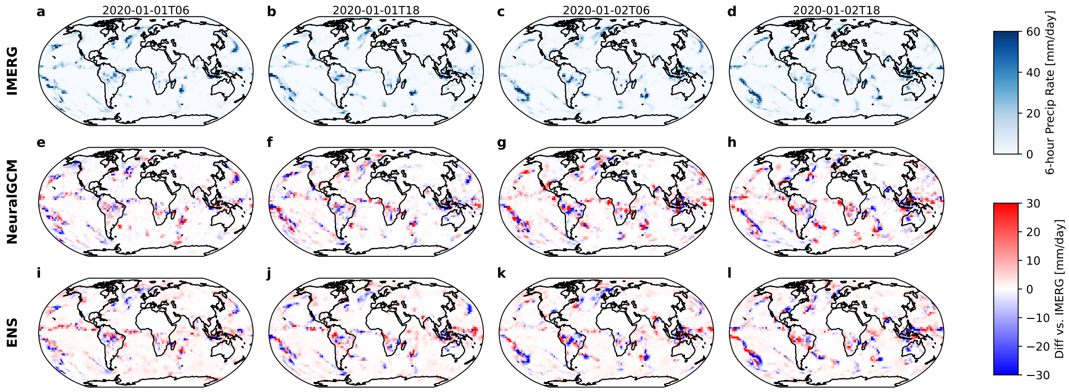

Figure S3: Comparison of 6-hour accumulated precipitation forecasts against IMERG observations. The top row (a-d) shows the observed precipitation rate from IMERG. The middle row (e-h) and bottom row (i-l) show the forecast error (Model - IMERG) for the first ensemble member of NeuralGCM and ECMWF ENS, respectively. Both forecasts were initialized at 01-01-2020T00 UTC. Timestamps indicate the end of the 6-hour accumulation period.

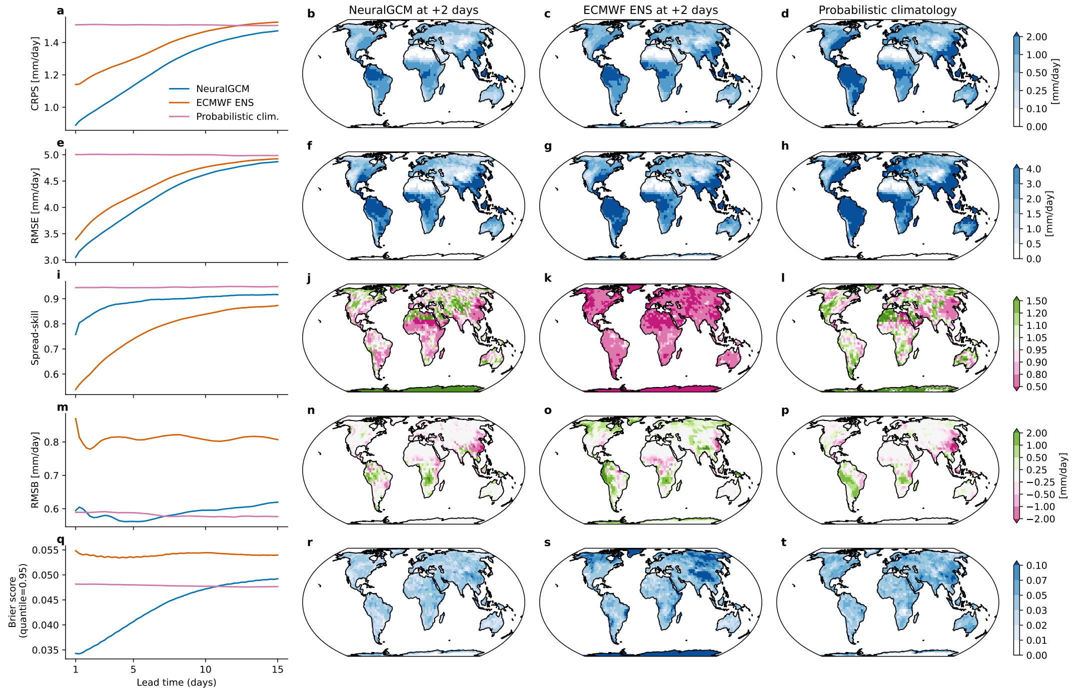

Figure S4: Precipitation forecasting accuracy scores for 24-hour accumulated precipitation over land, evaluated against IMERG. This figure is similar to Fig. 2, but the evaluation here is restricted to land areas.

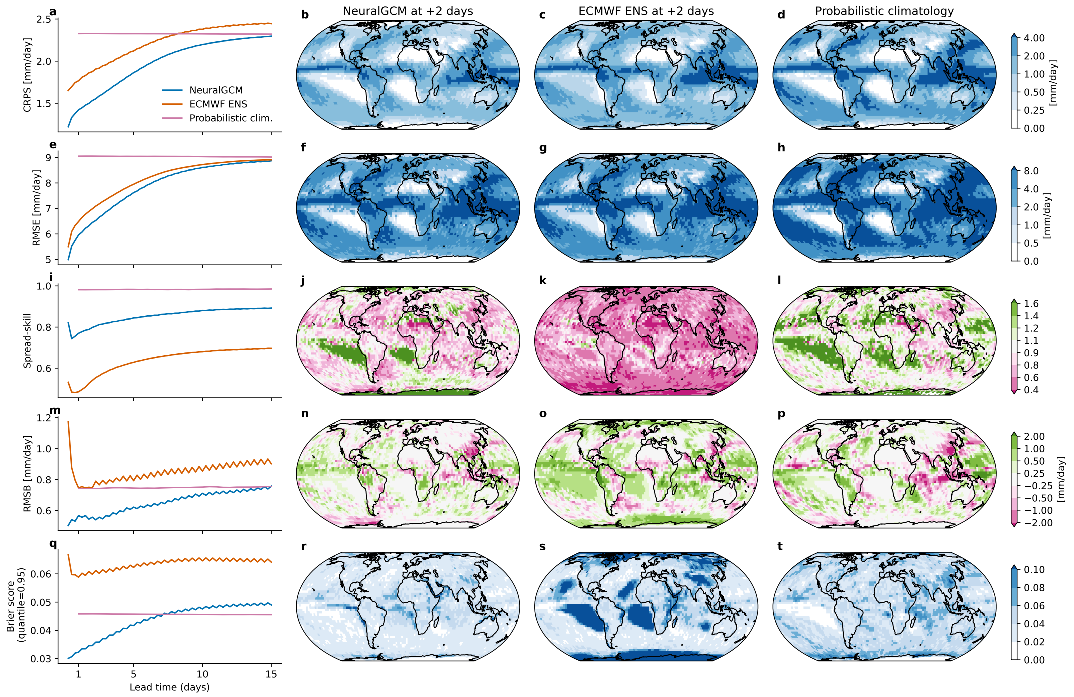

Figure S5: Precipitation forecasting accuracy scores for 6-hour accumulated precipitation over land, evaluated against IMERG. This figure is similar to Fig. 2, but the evaluation here is done on 6-hour accumulations.

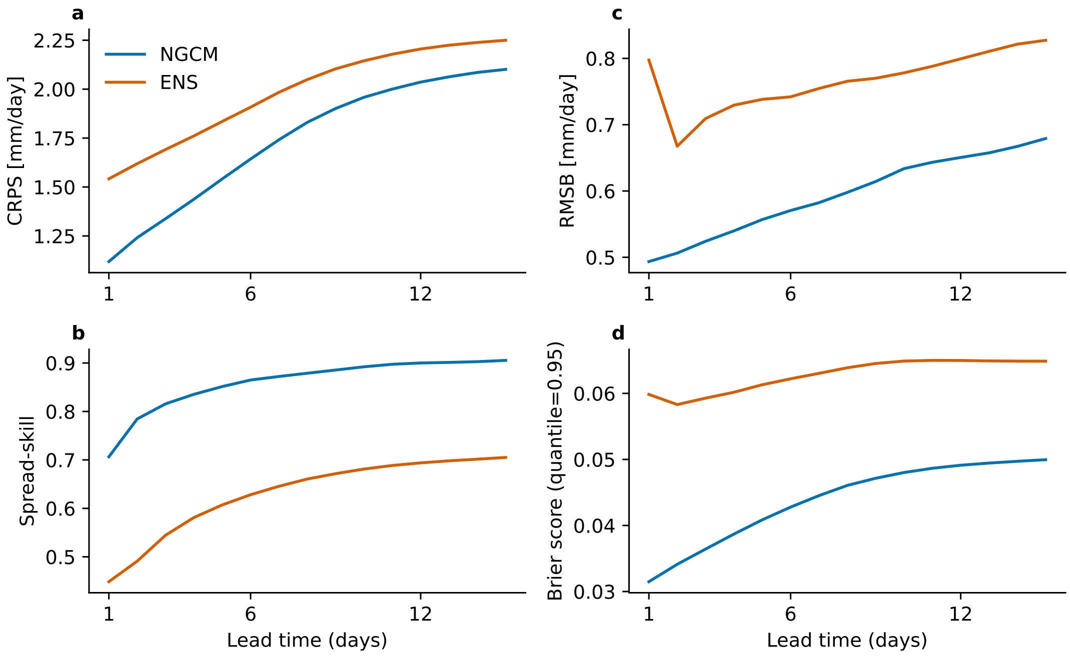

Figure S6: Precipitation forecasting accuracy scores for 24-hour accumulated precipitation, evaluated against GPCP. Area-weighted mean, calculated over all longitudes and latitudes between $-60°$ to $60°$ for: (a) Continuous Ranked Probability Score (CRPS). (b) Spread-skill ratio. (c) Root-mean-square bias (RMSB). (d) Brier score (0.95 quantile). Comparisons are shown for NeuralGCM and ECMWF ensemble.

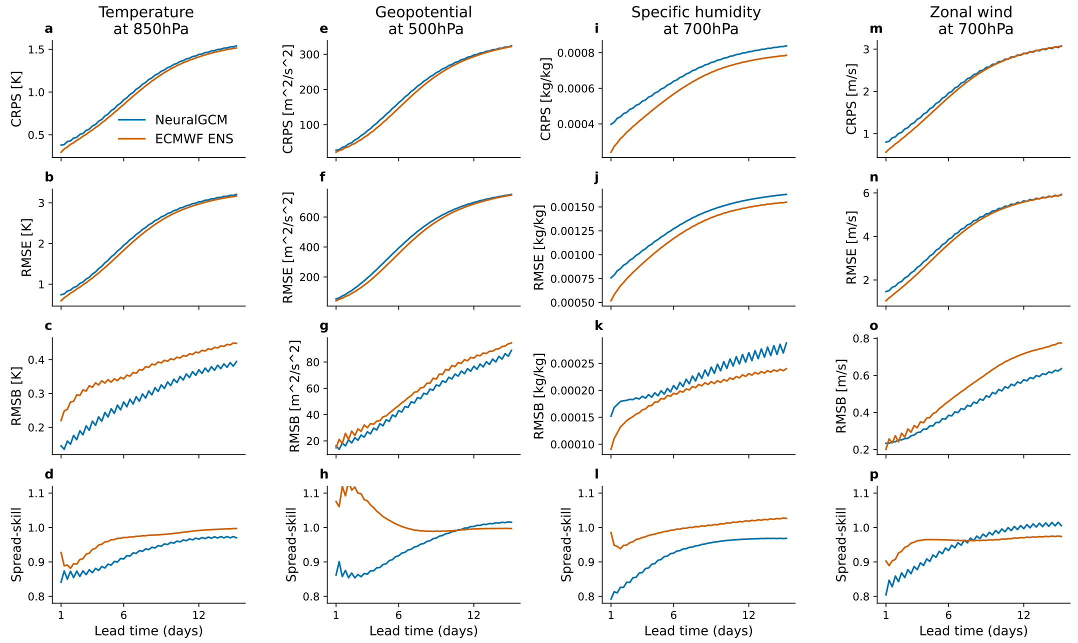

Figure S7: Weather forecasting accuracy scores for NeuralGCM and the ECMWF ENS for various atmospheric variables. Rows show different skill metrics: (1) Continuous Ranked Probability Score (CRPS), (2) ensemble mean root-mean-square error (RMSE), (3) root-mean-square bias (RMSB), and (4) spread-skill ratio. Columns show different variables: (a-d) temperature at 850 hPa, (e-h) geopotential at 500 hPa, (i-l) specific humidity at 700 hPa, and (m-p) zonal wind at 700 hPa. NeuralGCM is compared with ERA5 as the ground truth, whereas ECMWF-ENS is compared with the ECMWF operational analysis (that is, HRES at 0-hour lead time), to avoid penalizing the operational forecasts for different biases than ERA5.

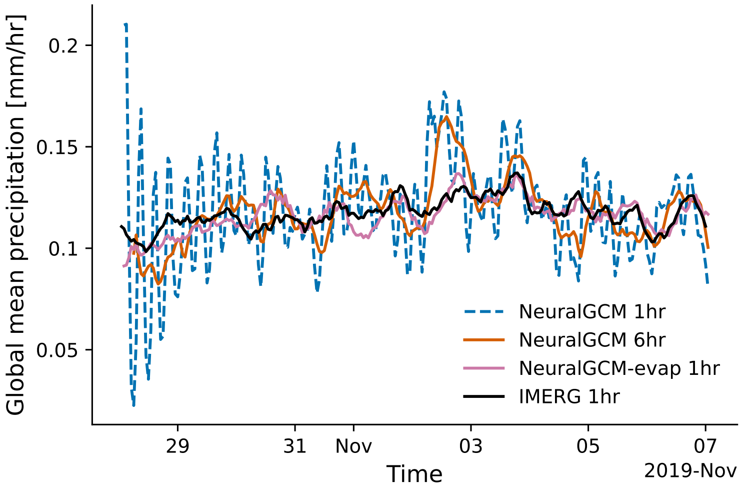

Figure S8: Global mean precipitation for 10-day forecasts. Time series of global mean precipitation for NeuralGCM at 1-hour and 6-hour frequencies, IMERG at 1-hour frequency, and NeuralGCM-evap at 1-hour frequency. NeuralGCM exhibits unrealistic fluctuations at frequencies higher than 6-hourly (see also Fig. 6 in the manuscript), while NeuralGCM-evap does not (see also Fig. S33). NeuralGCM forecasts were initialized on October 28, 2019.

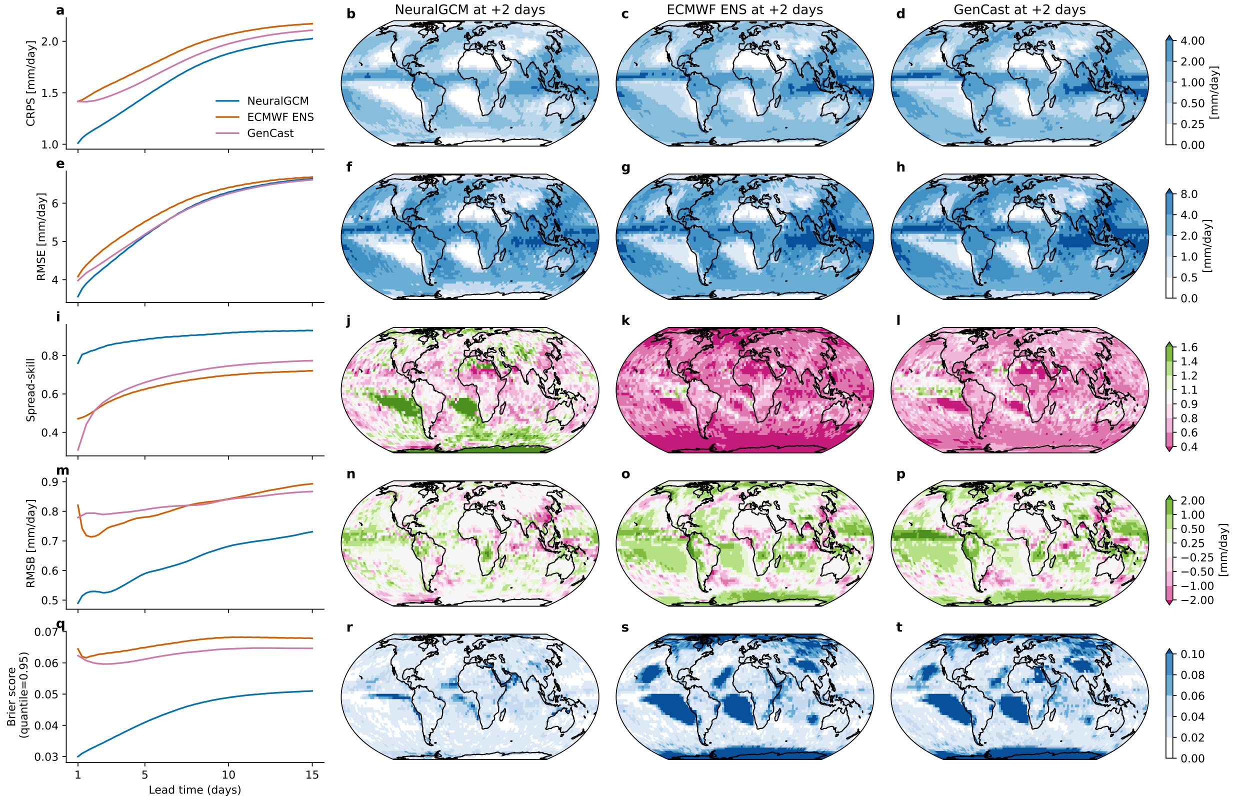

Figure S9: Precipitation forecasting accuracy scores for 24-hour accumulated precipitation from NeuralGCM, ENS, and GenCast (*54*), evaluated against IMERG. This figure is an extension of Fig. 2 to include GenCast. Note: This is not a direct "apples-to-apples" comparison, as GenCast was trained on ERA5 while NeuralGCM was trained on IMERG. The results should not be interpreted as a definitive measure of forecast skill, but rather as an illustration of the large differences between the ERA5 and IMERG precipitation datasets. GenCast was regridded to a 128x64 grid prior to evaluation.

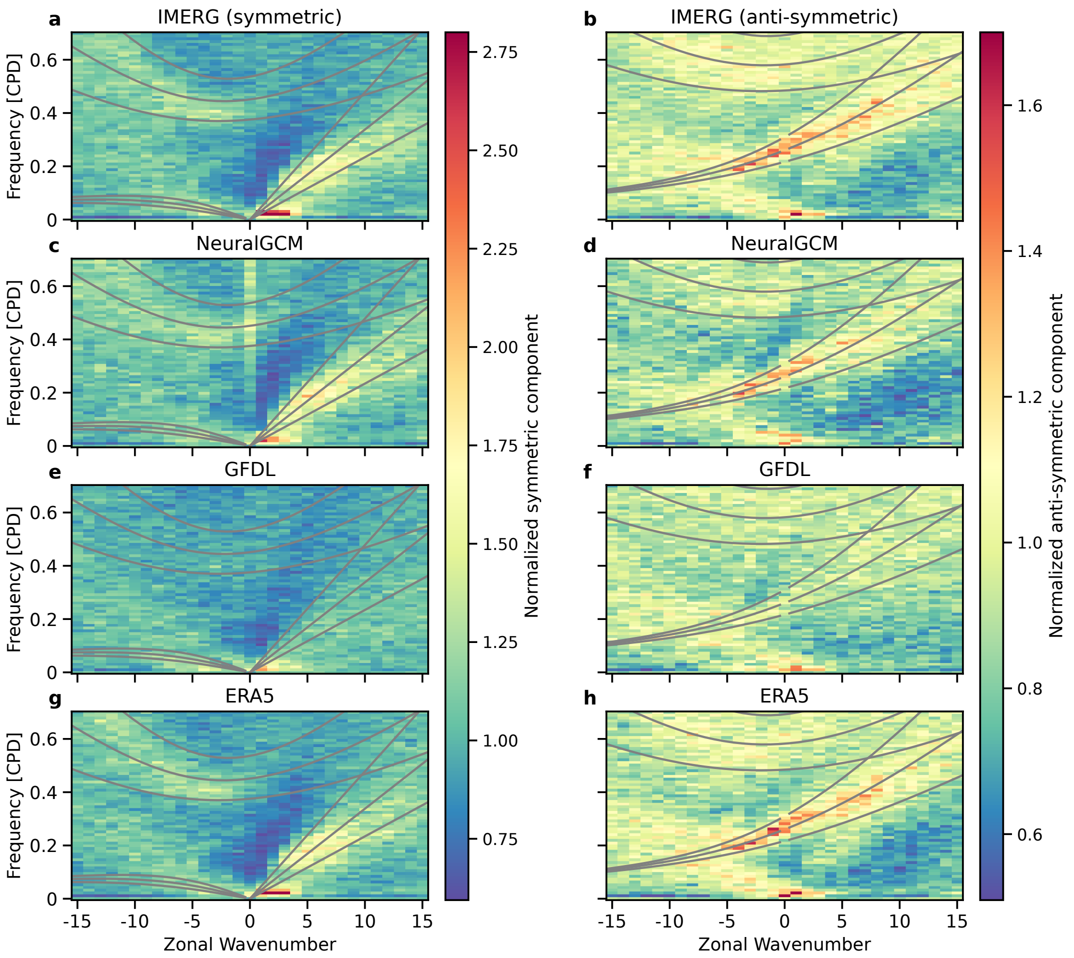

Figure S10: Space-time spectra of precipitation for IMERG, NeuralGCM, GFDL (AMIP run), and ERA5 (2002–2014). The Wheeler-Kiladis diagrams were constructed using 96-day windows with a 60-day overlap. To highlight the dominant wave modes, the power spectrum was normalized by a smoothed background spectrum, which was estimated by repeatedly convolving the spectrum in the frequency dimension (separately for positive and negative frequencies).

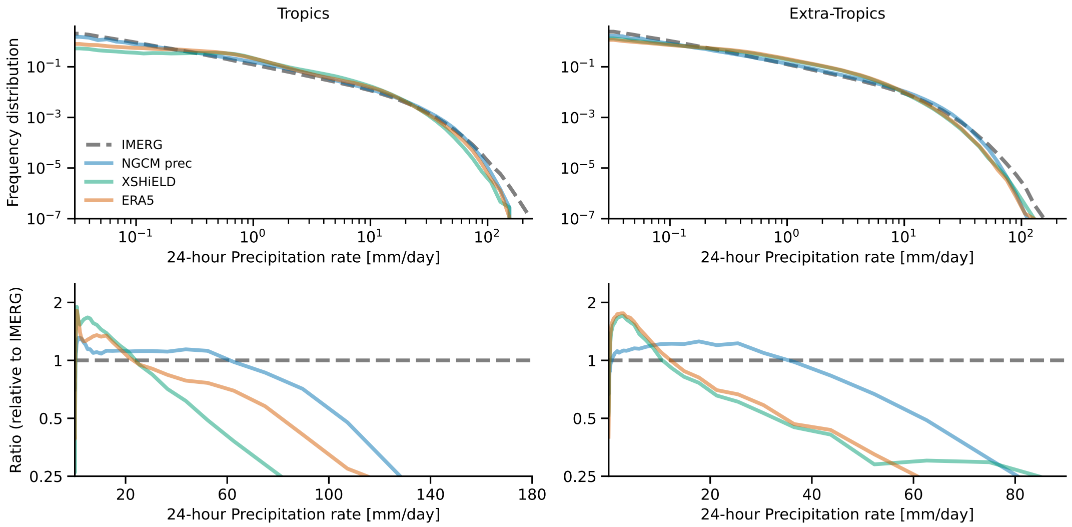

Figure S11: Precipitation rate distributions for IMERG, NeuralGCM, ERA5 and X-SHEiLD. Frequency distribution of 24-hourly precipitation rate for (a) tropics (latitudes -20 to 20) and (b) extra-tropics (latitudes 30 to 70 in both hemispheres) and the relative distribution (normalized by the IMERG value) for (c) tropics and (d) extra-tropics. Distributions for all models are calculated from the dates available in X-SHEiLD run (January 18th 2020 to January 17th 2021). NeuralGCM model was initialized on 2001-12-27. All models coarsened to 2.8° resolution.

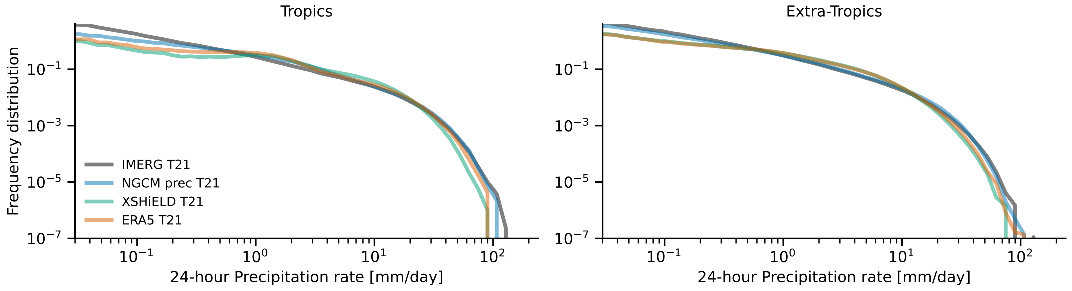

Figure S12: Precipitation rate distributions for IMERG, NeuralGCM, ERA5 and X-SHEiLD coarsened to 5.6° resolution. This figure is similar to fig. S11 (a-b), but all models were coarse grained to 5.6° resolution.

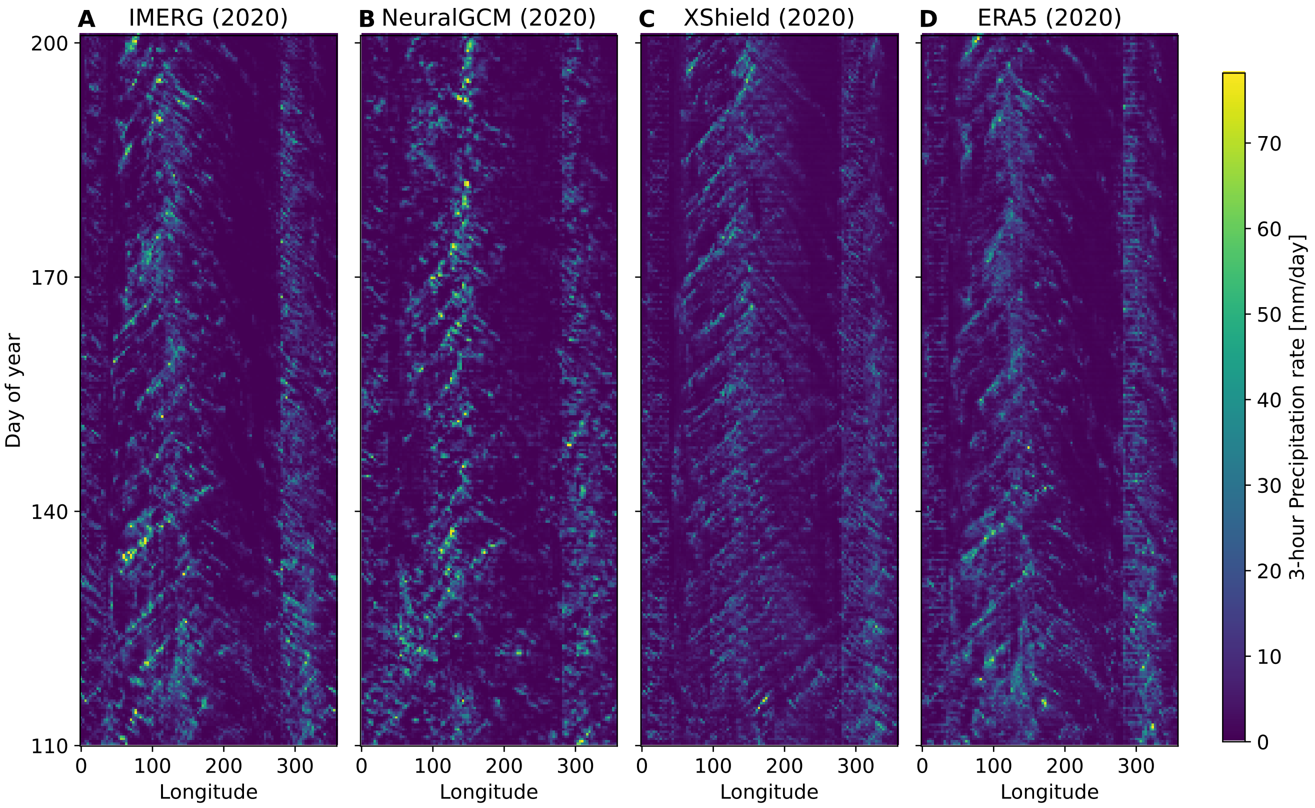

Figure S13: Hovmoller tropical precipitation diagram for different models. Precipitation is averaged between latitudes $-5°$ and $5°$. IMERG, NeuralGCM, X-SHiELD and ERA5 for 91 days starting on April 20, 2020. NeuralGCM run shown was initialized on December 27 2001. All models were coarse-grained to $2.8°$ before plotting. This figure is similar to Fig. 3 but is plotted for 2020, a hold-out year on which NeuralGCM was not trained, and a period for which X-SHiELD data were available.

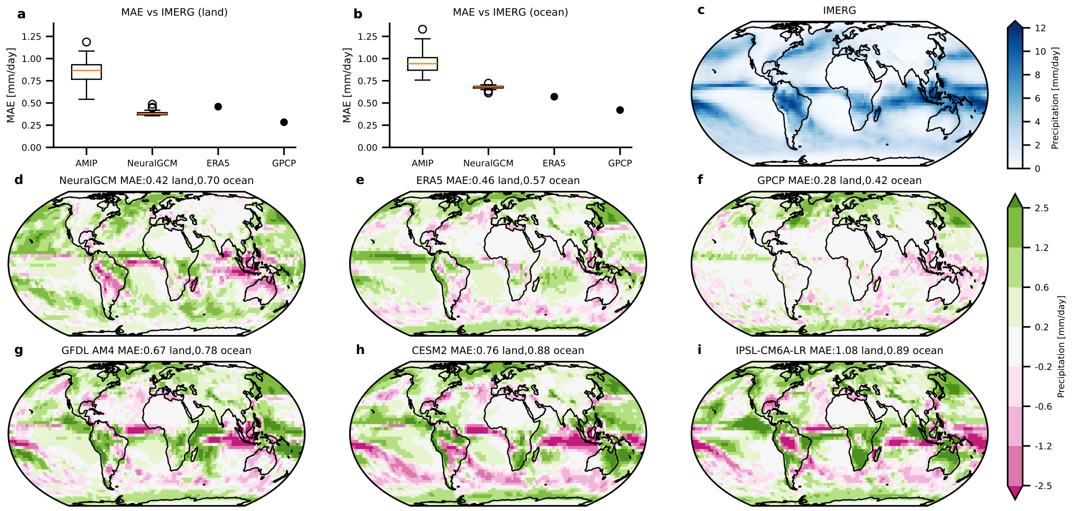

Figure S14: Mean bias in precipitation averaged over 2002–2014 for December-January-February (same as Fig. 4 but for DJF)

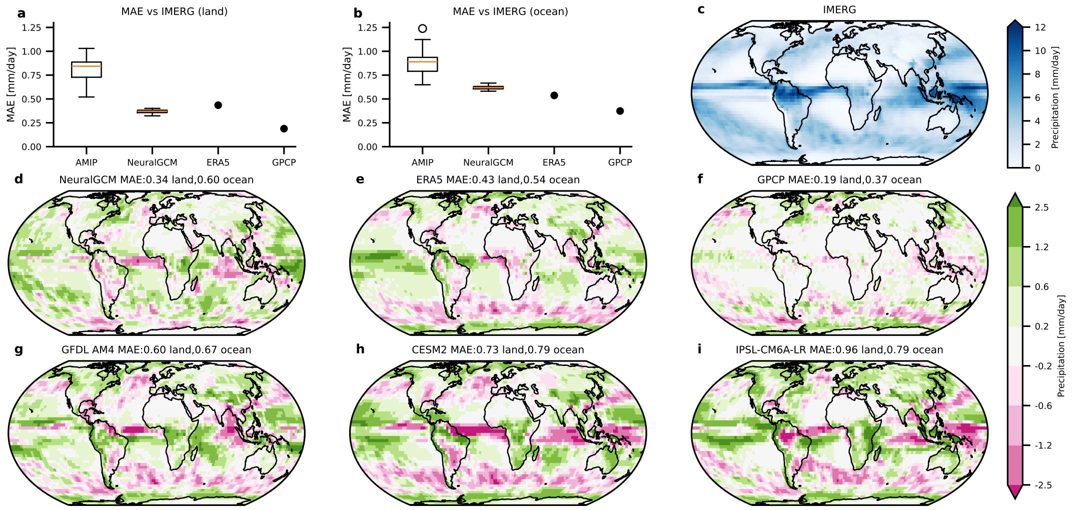

Figure S15: Mean bias in precipitation averaged over 2002–2014 for March-April-May (same as Fig. 4 but for MAM)

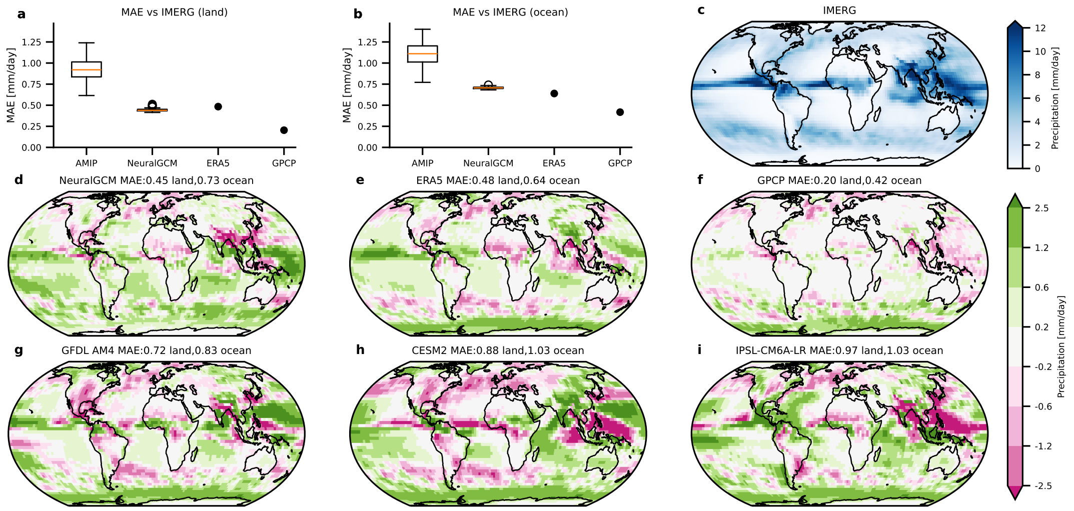

Figure S16: Mean bias in precipitation averaged over 2002–2014 for June-July-August (same as Fig. 4 but for JJA)

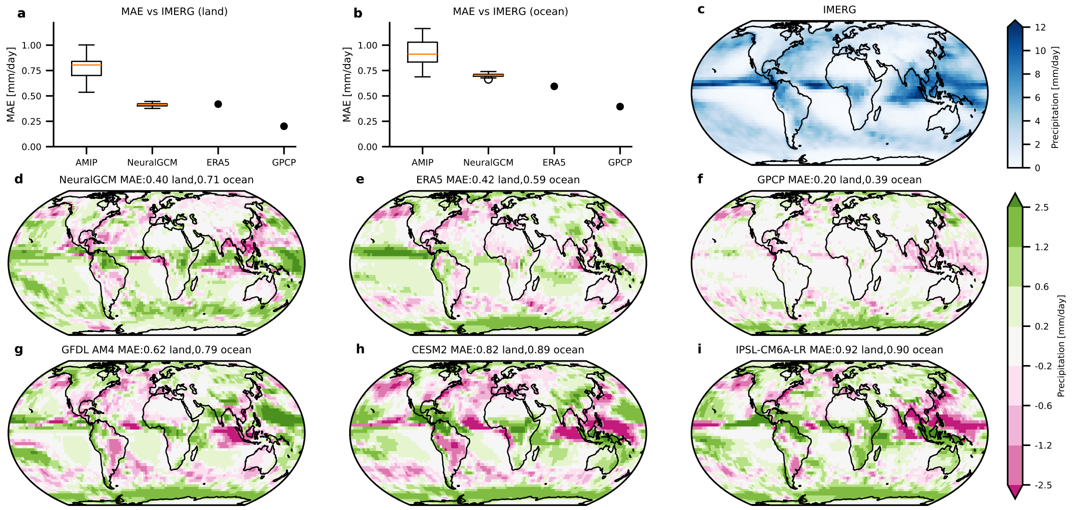

Figure S17: Mean bias in precipitation averaged over 2002–2014 for September-October-November (same as Fig. 4 but for SON)

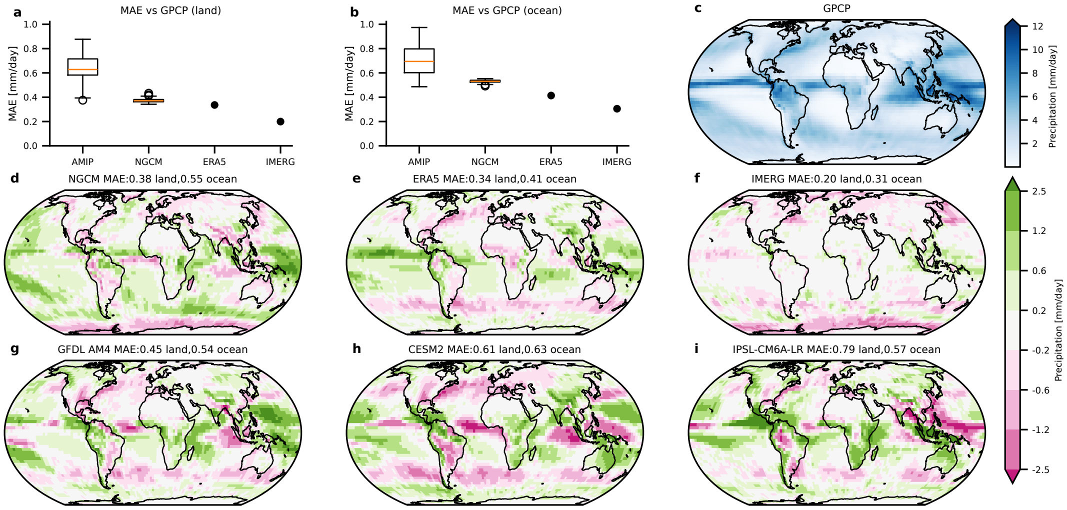

Figure S18: Mean bias in precipitation averaged over 2002–2014 but using GPCP (*40*) as a baseline (same as Fig. 4 but using GPCP as a baseline)

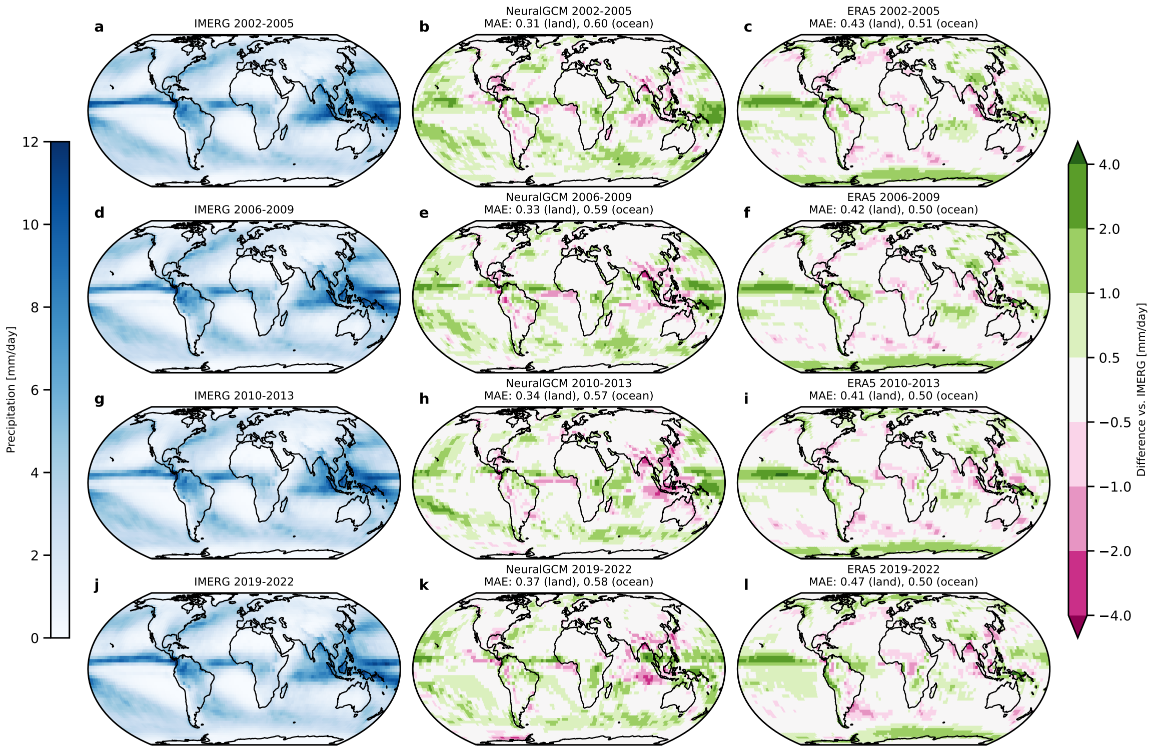

Figure S19: Comparison of mean precipitation bias across four-year periods. This figure evaluates the mean precipitation bias of NeuralGCM and ERA5 relative to IMERG observations across different four-year intervals. The analysis includes periods within the NeuralGCM training data (2002-2013, top three rows) and a holdout period on which the model was not trained (2019–2022, bottom row) to assess the model's generalization. The left column (a,d,g,j) displays the mean precipitation from the IMERG reference dataset. The middle column (b,e,h,k) shows the bias for NeuralGCM, while the right column (c,f,i,l) shows the bias for ERA5. For each bias map, the Mean Absolute Error (MAE) relative to IMERG is provided for land and ocean regions. The results show that the bias in the hold-out years is comparable to the training years. Over land, the bias for 2019-2022 is slightly larger, but we note that ERA5 exhibits its largest land bias in this same period. Over the ocean, the bias is similar across all years.

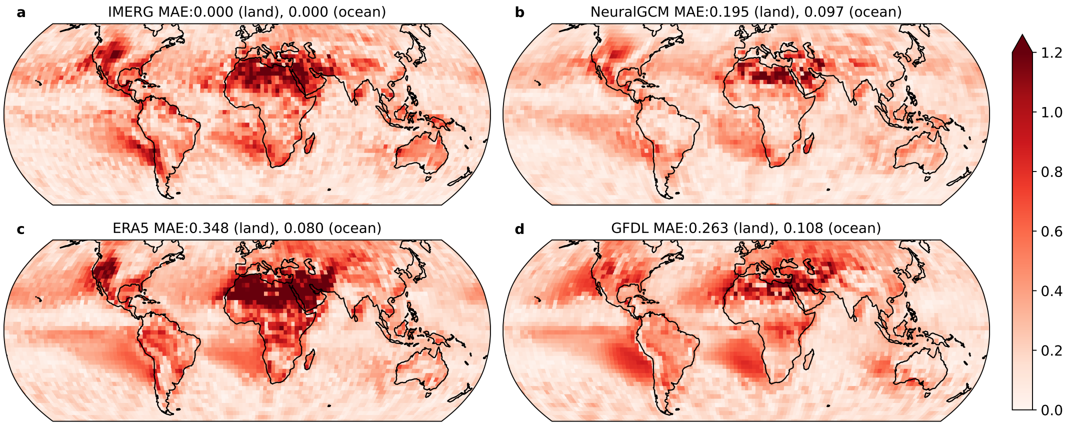

Figure S20: Diurnal harmonic amplitude of summertime precipitation (2002-2014). The plots show the diurnal amplitude ratio (amplitude divided by mean precipitation) used for the masking in Fig. 6 for (a) IMERG, (b) NeuralGCM, (c) ERA5, and (d) the GFDL AMIP simulation. Summertime is defined as July in the Northern Hemisphere and January in the Southern Hemisphere. Each panel's title includes the Mean Absolute Error (MAE) relative to IMERG for both land and ocean.

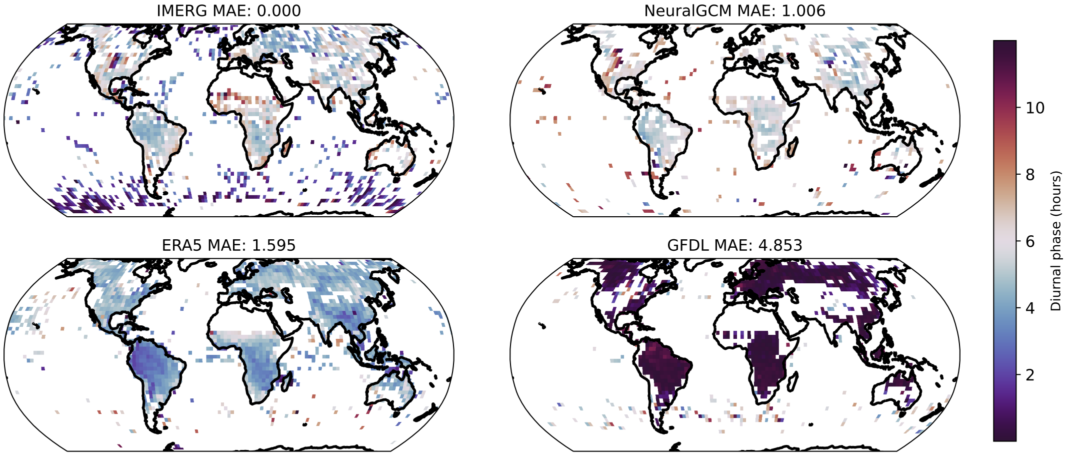

Figure S21: Semi-diurnal Cycle of Summertime Precipitation (2002-2014). Same as Fig. 6 but for the semi-diurnal phase.

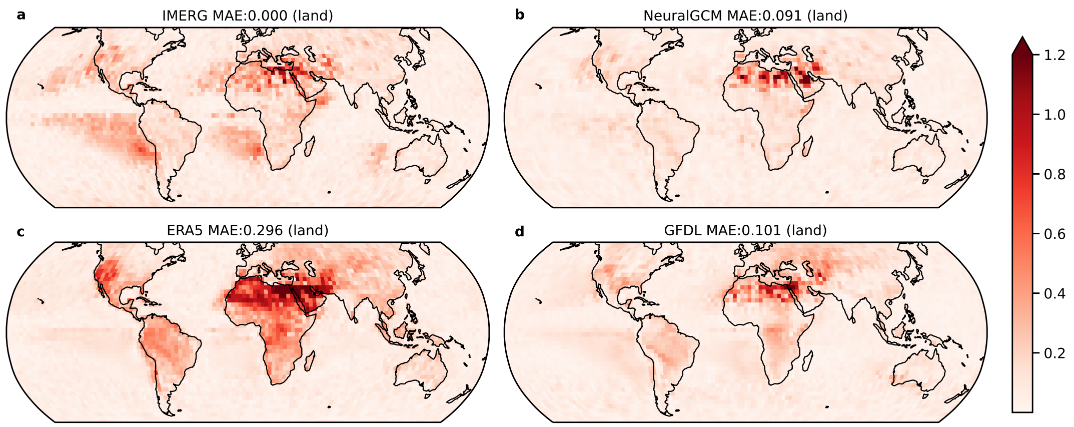

Figure S22: Semi-diurnal harmonic amplitude of summertime precipitation (divided by the monthly mean precipitation; 2002-2014).

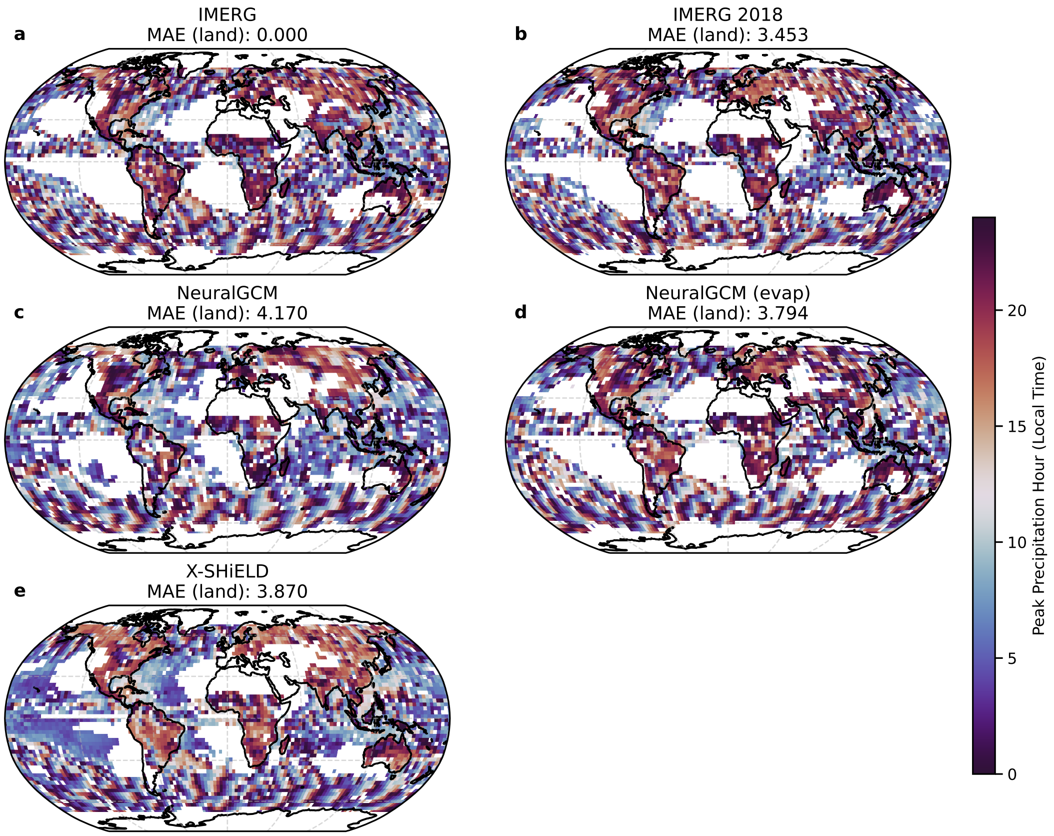

Figure S23: Diurnal Cycle of Summertime Precipitation (January 18, 2020, to January 17, 2021). This figure is similar to Fig. 6, but all datasets (except IMERG 2018) are evaluated for the period from January 18, 2020, to January 17, 2021. Panels show: (a) the reference data from IMERG for this period; (b) IMERG data from a different year (2018) to provide a baseline for interannual variability; (c) NeuralGCM; (d) NeuralGCM-evap; and (e) X-SHIELD. The models show similar performance during this period. NeuralGCM has a slightly larger bias than X-SHIELD, while NeuralGCM-evap has a slightly smaller bias. However, because all model biases are comparable in magnitude to the interannual variability between different years of IMERG data, it is challenging to definitively determine which model performs best.

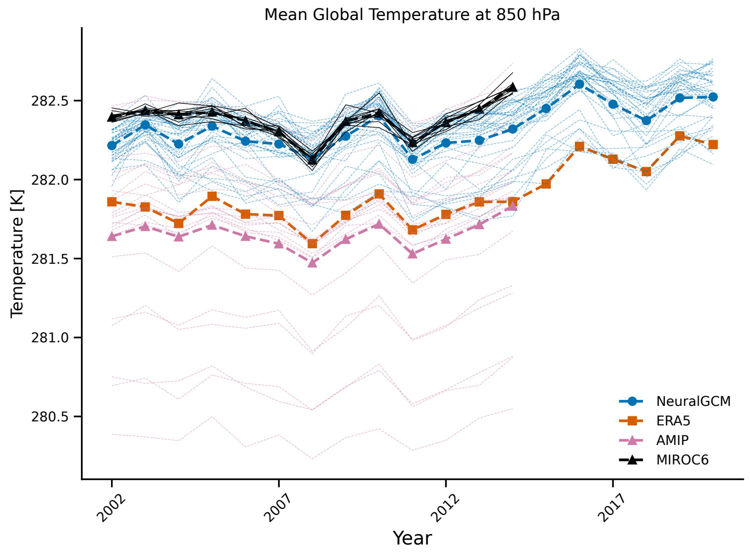

Figure S24: Global mean temperature for ERA5, NeuralGCM, CMIP6 AMIP runs, and 10 members of MIROC6 AMIP runs. Bold lines show the NeuralGCM ensemble mean, and AMIP models mean. AMIP models used in this plot are described in the methods.

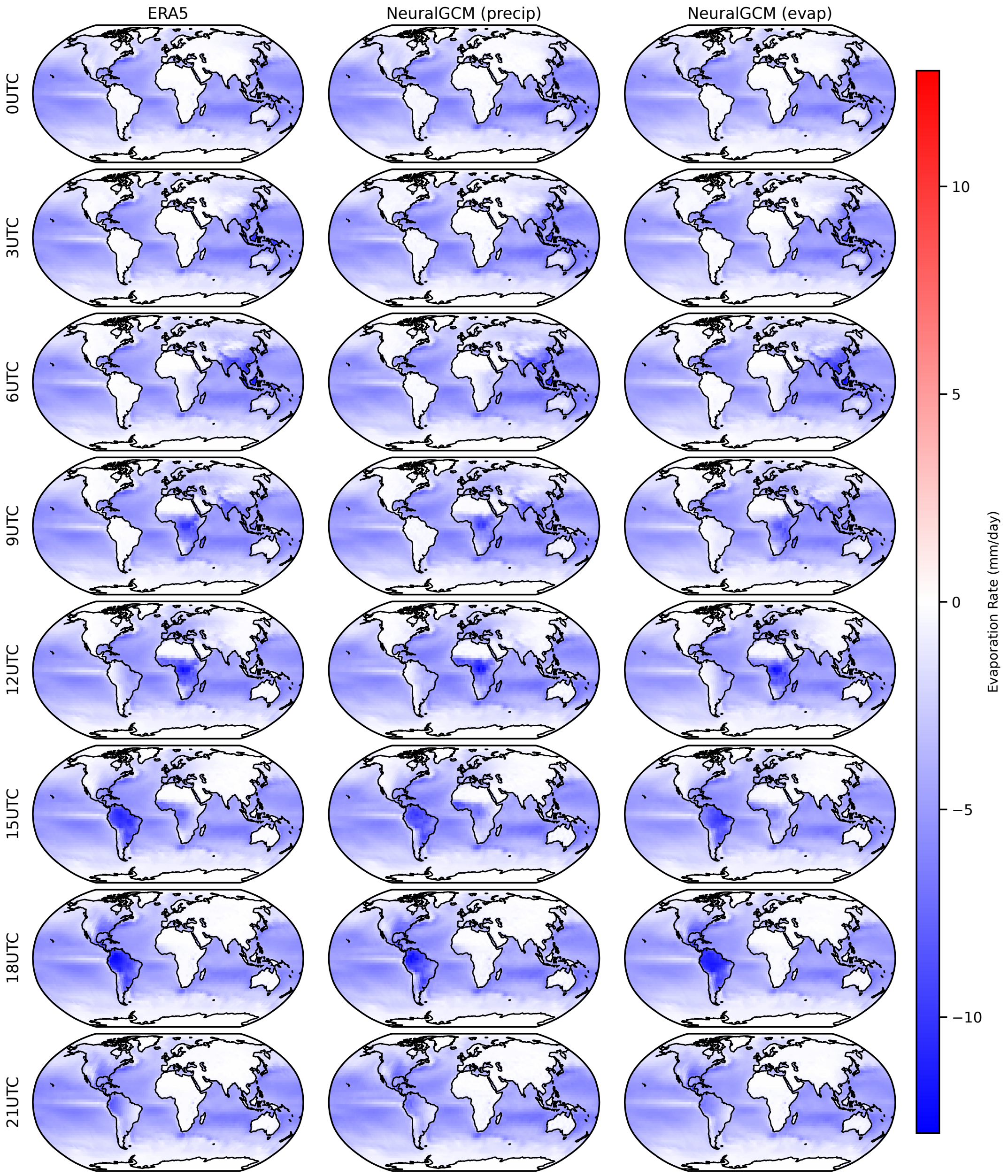

Figure S25: Diurnal cycle of hourly mean evaporation rate for ERA5 and two NeuralGCM configurations: (1) precipitation predicted, evaporation diagnosed; (2) evaporation predicted, precipitation diagnosed. Evaporation is averaged over 2020 (day 2 of the NeuralGCM simulations, initialized on December 27, 2001).

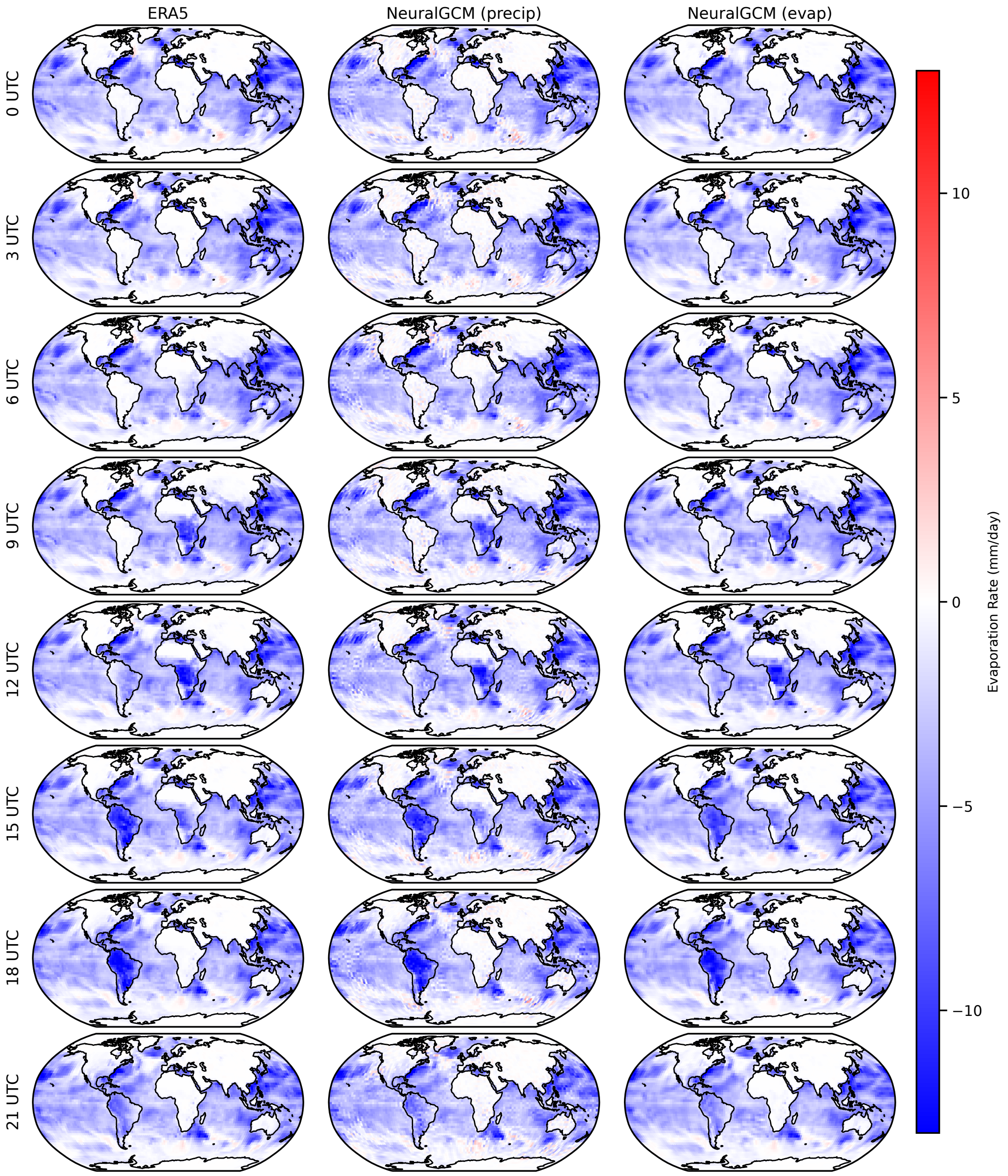

Figure S26: Diurnal cycle of instantaneous evaporation rate for ERA5 and two NeuralGCM configurations: (1) precipitation predicted, evaporation diagnosed; (2) evaporation predicted, precipitation diagnosed. Shown for December 28, 2001 (day 2 of the NeuralGCM simulations, initialized on December 27, 2001).

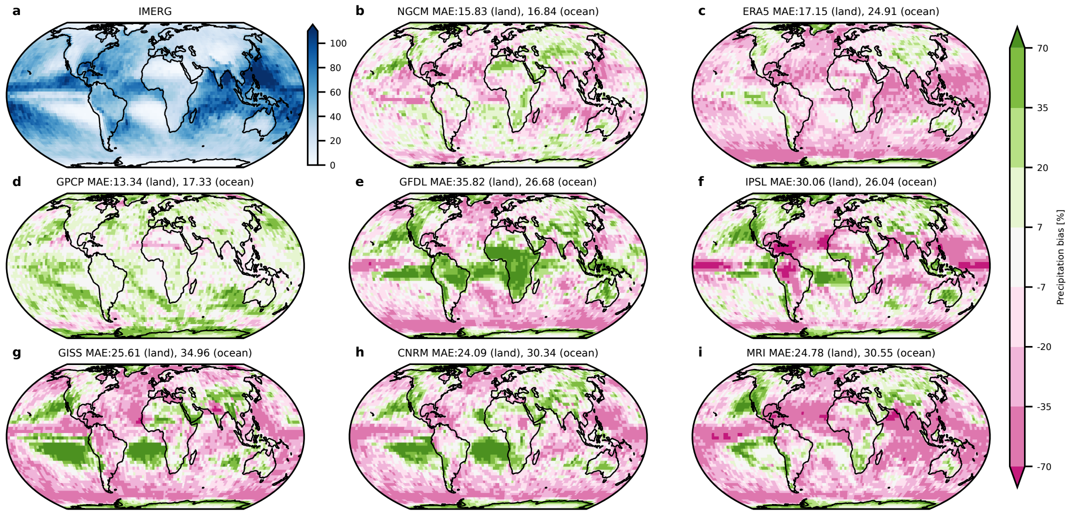

Figure S27: Percent mean absolute error in annual maximum daily precipitation (Rx1day) averaged over 2002–2014. (a) IMERG (*37*) in mm/day. (b–i) Percent error in Rx1day for NeuralGCM, ERA5, GPCP (*40*), and various CMIP6 historical simulations, relative to IMERG. The percent error is calculated as $(model - IMERG)/\max(\text{IMERG}, 20)$ to de-emphasize errors in regions where Rx1day is lower than 20 mm/day. Global mean absolute error (in percent) is shown for land and ocean regions.

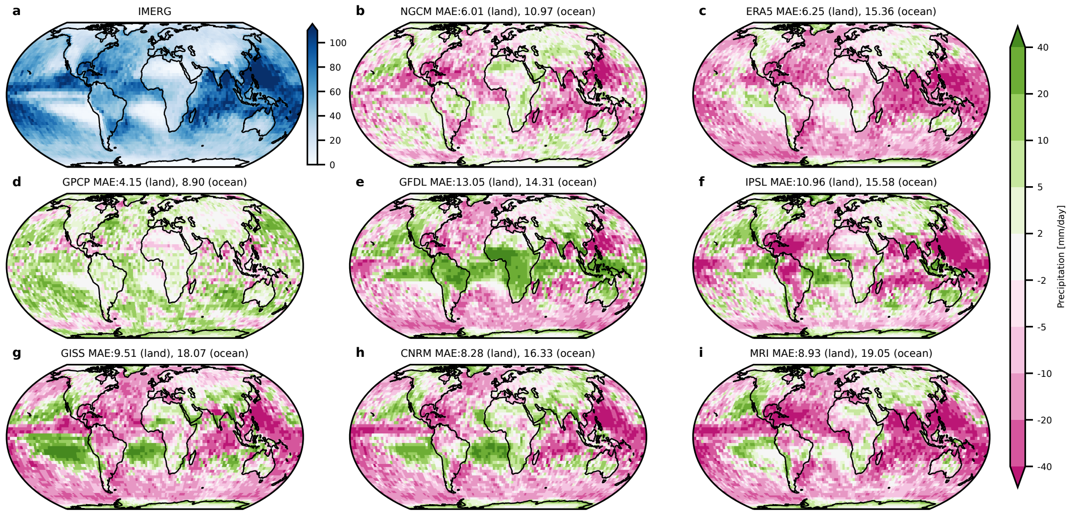

Figure S28: jy99.9th percentile of daily precipitation averaged over 2002–2014. (a) IMERG (*37*). (b–i) Bias in the 99.9th percentile of precipitation for NeuralGCM, ERA5, GPCP (*40*), and various CMIP6 historical simulations, relative to IMERG. Mean absoulute error (MAE) vs. IMERG is shown for land and ocean regions.

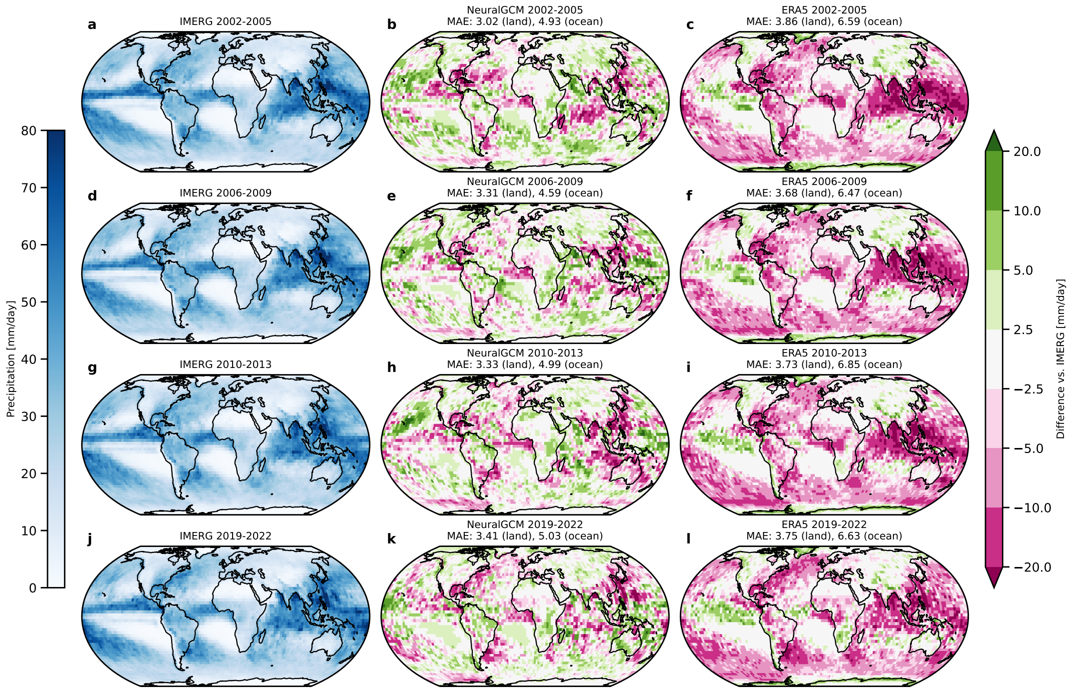

Figure S29: jyBias in the 99th percentile of daily precipitation across four-year periods. The figure compares NeuralGCM and ERA5 against IMERG observations for periods within the training data (2002-2013, top three rows) and a holdout period (2019–2022, bottom row). The left column (a,d,g,j) shows the 99th percentile precipitation for IMERG. The middle (b,e,h,k) and right (c,f,i,l) columns show the corresponding bias for NeuralGCM and ERA5, respectively.

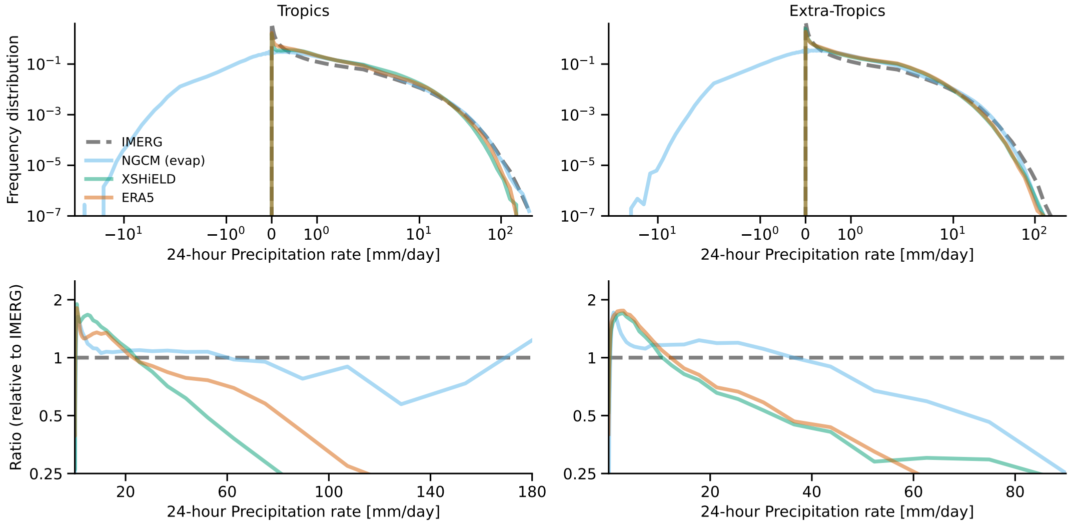

Figure S30: Precipitation rate frequency distributions for IMERG, NeuralGCM (evap), ERA5 and X-SHjyiELD. Like fig. S11 but showing NeruralGCM-evap model that predicts evaporation and diagnose precipitation. The distribution shows that NeruralGCM-evap produces frequent negative precipitation.

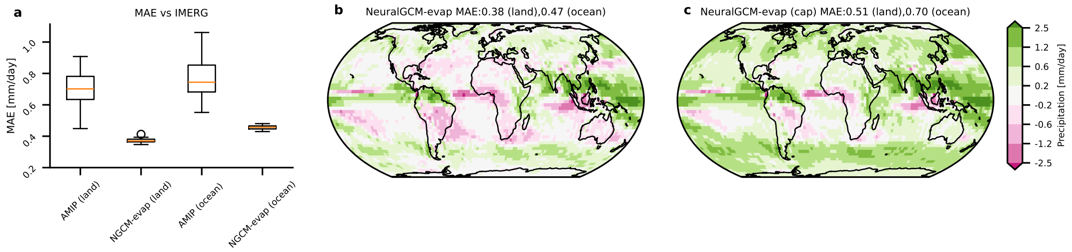

Figure S31: Mean absolute error (MAE) for precipitation averaged over 2002–2014 for the NeuralGCM-evap model. This figure is similar to Fig. 4 but shows results for: (a, b) the NeuralGCM-evap model, which predicts evaporation and diagnoses precipitation; and (c) the NeuralGCM-evap-cap model, a variant of NeuralGCM-evap where negative precipitation values are set to zero. Global MAE is shown for land and ocean regions.

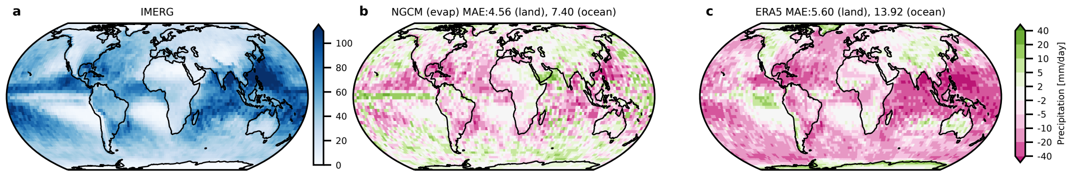

Figure S32: Annual maximum daily precipitation (Rx1day) averaged over 2002–2014 for NeuralGCM-evap model. This figure is similar to Fig. 5c-e but shows results for (b) NeuralGCM-evap model which predicts evaporation and diagnose precipitation. Global MAE is shown for land and ocean regions.

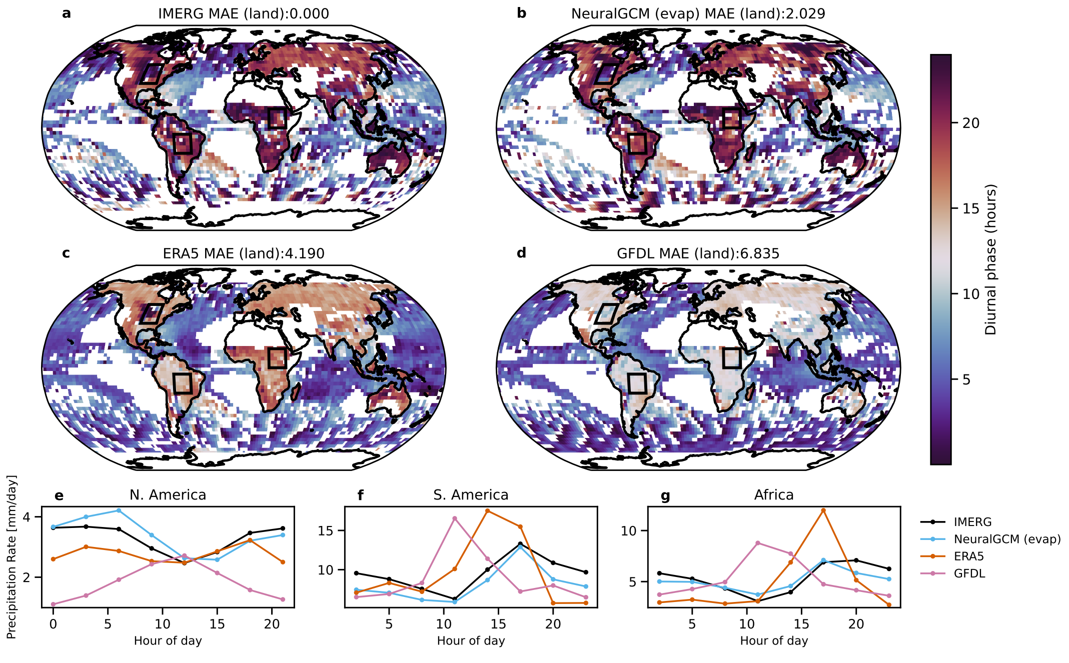

Figure S33: Diurnal Cycle of Summertime Precipitation (2002-2014) for NeuralGCM-evap. Like Fig. 6 but showing results for NeuralGCM-evap model which predicts evaporation and diagnose precipitation.

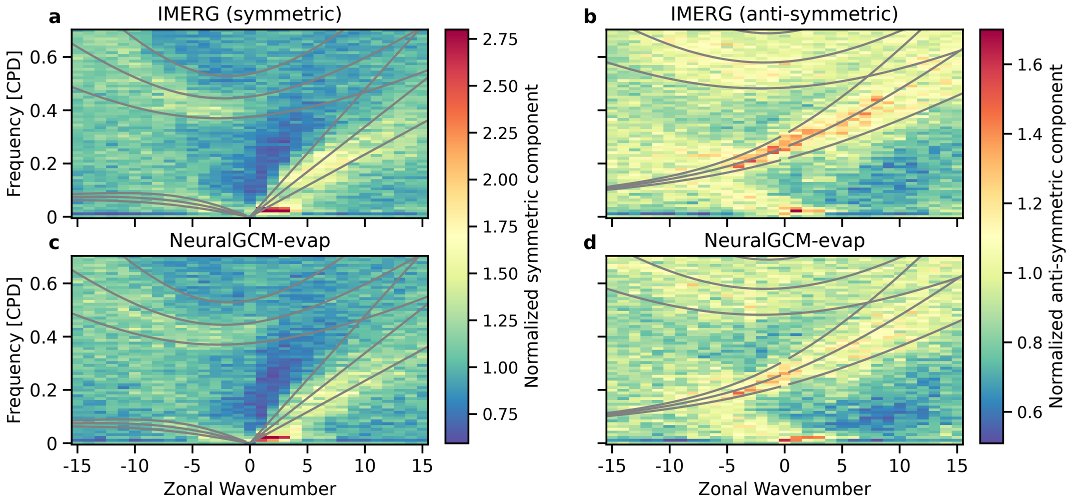

Figure S34: Space-Time Spectra of precipitation for IMERG and NeuralGCM-evap (2002-2014). Like fig. S10 but for NeuralGCM-evap model which predicts evaporation and diagnose precipitation.

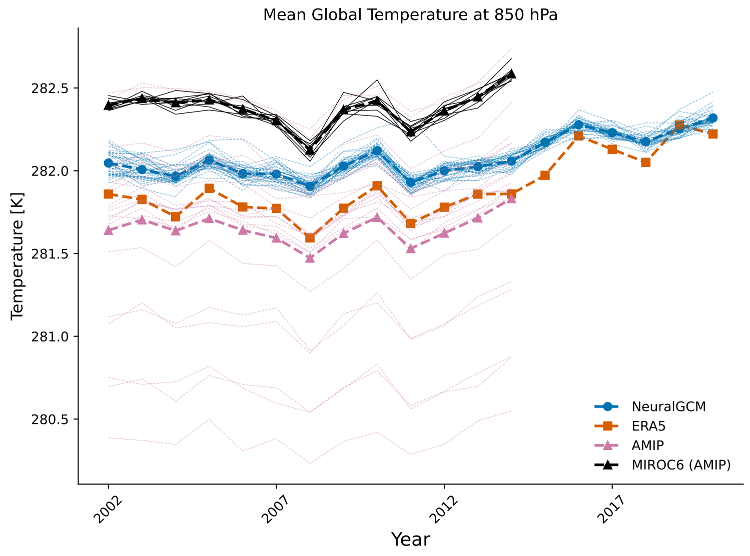

Figure S35: Global mean temperature for ERA5, NeuralGCM-evap, CMIP6 AMIP runs, and 10 members of MIROC6 AMIP runs. Same as fig. S24, but showing results for NeuralGCM-evap model, which predicts evaporation and diagnoses precipitation

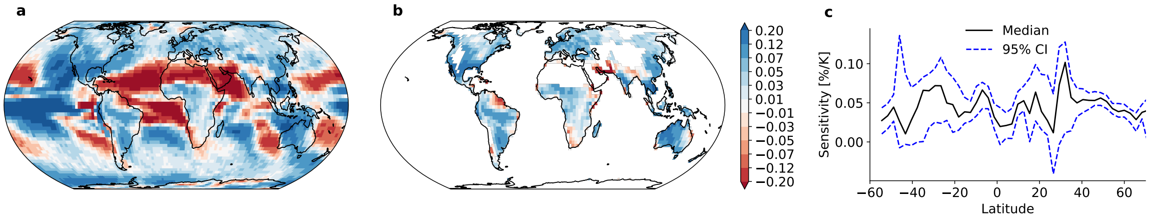

Figure S36: Sensitivity of annual-maximum daily precipitation (Rx1day) to changes in global mean temperature, calculated from NeuralGCM large ensemble runs over 2002–2021. (a) Sensitivity for all latitudes and longitudes, in units of % K^{-1}. (b) Same as panel (a), but showing values only over land. (c) Median sensitivity over land (solid line; dotted lines show the 95% confidence interval), averaged over longitudinal bands, as a function of global mean 850hPa temperature. Regions where Rx1day $<$ 20 mm/day where masked out. Sensitivity was derived using Theil-Sen regression of Rx1day against global mean temperature for each year and ensemble member in each grid box over the period 2002–2021. Confidence intervals were used from the Theil-Sen regression.

REFERENCES

1. L. Bock, A. Lauer, M. Schlund, M. Barreiro, N. Bellouin, C. Jones, G. Meehl, V. Predoi, M. Roberts, V. Eyring, Quantifying progress across different CMIP phases with the ESMValTool. *J. Geophys. Res. Atmos.* **125**, e2019JD032321 (2020).

2. T. Palmer, B. Stevens, The scientific challenge of understanding and estimating climate change. *Proc. Natl. Acad. Sci. U.S.A.* **116**, 24390–24395 (2019).

3. C. Tebaldi, K. Debeire, V. Eyring, E. Fischer, J. Fyfe, P. Friedlingstein, R. Knutti, J. Lowe, B. O'Neill, B. Sanderson, D. van Vuuren, K. Riahi, M. Meinshausen, Z. Nicholls, K. B. Tokarska, G. Hurtt, E. Kriegler, J.-F. Lamarque, G. Meehl, R. Moss, S. E. Bauer, O. Boucher, V. Brovkin, Y.-H. Byun, M. Dix, S. Gualdi, H. Guo, J. G. John, S. Kharin, Y. H. Kim, T. Koshiro, L. Ma, D. Olivié, S. Panickal, F. Qiao, X. Rong, N. Rosenbloom, M. Schupfner, R. Séférian, A. Sellar, T. Semmler, X. Shi, Z. Song, C. Steger, R. Stouffer, N. Swart, K. Tachiiri, Q. Tang, H. Tatebe, A. Voldoire, E. Volodin, K. Wyser, X. Xin, S. Yang, Y. Yu, T. Ziehn, Climate model projections from the scenario model intercomparison project (ScenarioMIP) of CMIP6. *Earth Syst. Dynam.* **12**, 253–293 (2021).

4. A. Dai, Precipitation characteristics in eighteen coupled climate models. *J. Clim.* **19**, 4605–4630 (2006).

5. S. Fiedler, T. Crueger, R. D'Agostino, K. Peters, T. Becker, D. Leutwyler, L. Paccini, J. Burdanowitz, S. A. Buehler, A. U. Cortes, T. Dauhut, D. Dommenget, K. Fraedrich, L. Jungandreas, N. Maher, A. K. Naumann, M. Rugenstein, M. Sakradzija, H. Schmidt, F. Sielmann, C. Stephan, C. Timmreck, X. Zhu, B. Stevens, Simulated tropical precipitation assessed across three major phases of the coupled model intercomparison project (CMIP). *Mon. Weather Rev.* **148**, 3653–3680 (2020).

6. S. Tang, P. Gleckler, S. Xie, J. Lee, M.-S. Ahn, C. Covey, C. Zhang, Evaluating the diurnal and semidiurnal cycle of precipitation in CMIP6 models using satellite-and ground-based observations. *J. Clim.* **34**, 3189–3210 (2021).

7. K. E. Trenberth, A. Dai, R. M. Rasmussen, D. B. Parsons, The changing character of precipitation. *Bull. Am. Meteorol. Soc.* **84**, 1205–1218 (2003).

8. C. B. Field, *Managing the Risks of Extreme Events and Disasters to Advance Climate Change Adaptation: Special Report of the Intergovernmental Panel on Climate Change* (Cambridge Univ. Press, 2012).

9. M. Wehner, P. Gleckler, J. Lee, Characterization of long period return values of extreme daily temperature and precipitation in the CMIP6 models: Part 1, model evaluation. *Weather Clim. Extrem.* **30**, 100283 (2020).

10. E. M. Wilcox, L. J. Donner, The frequency of extreme rain events in satellite rain-rate estimates and an atmospheric general circulation model. *J. Clim.* **20**, 53–69 (2007).

11. Intergovernmental Panel on Climate Change (IPCC), "Water cycle changes" in *Climate Change 2021—The Physical Science Basis: Working Group I Contribution to the Sixth Assessment Report of the Intergovernmental Panel on Climate Change* (Cambridge Univ. Press, 2023).

12. B. Stevens, M. Satoh, L. Auger, J. Biercamp, C. S. Bretherton, X. Chen, P. Düben, F. Judt, M. Khairoutdinov, D. Klocke, C. Kodama, L. Kornblueh, S.-J. Lin, P. Neumann, W. M. Putman, N. Röber, R. Shibuya, B. Vanniere, P. L. Vidale, N. Wedi, L. Zhou, DYAMOND: The DYnamics of the Atmospheric general circulation Modeled On Non-hydrostatic Domains. *Prog. Earth Planet. Sci.* **6**, 1–17 (2019).

13. J. Slingo, P. Bates, P. Bauer, S. Belcher, T. Palmer, G. Stephens, B. Stevens, T. Stocker, G. Teutsch, Ambitious partnership needed for reliable climate prediction. *Nat. Clim. Chang.* **12**, 499–503 (2022).

14. H.-Y. Ma, S. A. Klein, J. Lee, M.-S. Ahn, C. Tao, P. J. Gleckler, Superior daily and sub-daily precipitation statistics for intense and long-lived storms in global storm-resolving models. *Geophys. Res. Lett.* **49**, e2021GL096759 (2022).

15. Z. Feng, L. R. Leung, J. Hardin, C. R. Terai, F. Song, P. Caldwell, Mesoscale convective systems in DYAMOND global convection-permitting simulations. *Geophys. Res. Lett.* **50**, e2022GL102603 (2023).

16. S. Ravuri, K. Lenc, M. Willson, D. Kangin, R. Lam, P. Mirowski, M. Fitzsimons, M. Athanassiadou, S. Kashem, S. Madge, R. Prudden, A. Mandhane, A. Clark, A. Brock, K. Simonyan, R. Hadsell, N. Robinson, E. Clancy, A. Arribas, S. Mohamed, Skilful precipitation nowcasting using deep generative models of radar. *Nature* **597**, 672–677 (2021).

17. R. Lam, A. Sanchez-Gonzalez, M. Willson, P. Wirnsberger, M. Fortunato, F. Alet, S. Ravuri, T. Ewalds, Z. Eaton-Rosen, W. Hu, A. Merose, S. Hoyer, G. Holland, O. Vinyals, J. Stott, A. Pritzel, S. Mohamed, P. Battaglia, Learning skillful medium-range global weather forecasting. *Science* **382**, 1416–1421 (2023).

18. O. Watt-Meyer, G. Dresdner, J. McGibbon, S. K. Clark, B. Henn, J. Duncan, N. D. Brenowitz, K. Kashinath, M. S. Pritchard, B. Bonev, M. E. Peters, C. S. Bretherton, ACE: A fast, skillful learned global atmospheric model for climate prediction. arXiv:2310.02074 [physics.ao-ph] (2023).

19. N. Cresswell-Clay, B. Liu, D. Durran, A. Liu, Z. I. Espinosa, R. Moreno, M. Karlbauer, A deep learning earth system model for stable and efficient simulation of the current climate. arXiv:2409.16247 [physics.ao-ph] (2024).

20. J. Stock, J. Pathak, Y. Cohen, M. Pritchard, P. Garg, D. Durran, M. Mardani, N. Brenowitz, DiffObs: Generative Diffusion for Global Forecasting of Satellite Observations. arXiv:2404.06517 [physics.comp-ph] (2024).

21. J. P. Duncan, E. Wu, J.-C. Golaz, P. M. Caldwell, O. Watt-Meyer, S. K. Clark, J. McGibbon, G. Dresdner, K. Kashinath, B. Bonev, M. S. Pritchard, C. S. Bretherton, Application of the AI2 Climate Emulator to E3SMv2's global atmosphere model, with a focus on precipitation fidelity. *J. Geophys. Res. Mach. Learn. Comput.* **1**, e2024JH000136 (2024).

22. P. Gentine, M. Pritchard, S. Rasp, G. Reinaudi, G. Yacalis, Could machine learning break the convection parameterization deadlock? *Geophys. Res. Lett.* **45**, 5742–5751 (2018).

23. S. Rasp, M. S. Pritchard, P. Gentine, Deep learning to represent subgrid processes in climate models. *Proc. Natl. Acad. Sci. U.S.A.* **115**, 9684–9689 (2018).

24. J. Yuval, P. A. O'Gorman, Stable machine-learning parameterization of subgrid processes for climate modeling at a range of resolutions. *Nat. Commun.* **11**, 3295 (2020).

25. J. Yuval, P. A. O'Gorman, C. N. Hill, Use of neural networks for stable, accurate and physically consistent parameterization of subgrid atmospheric processes with good performance at reduced precision. *Geophys. Res. Lett.* **48**, e2020GL091363 (2021).

26. N. D. Brenowitz, T. Beucler, M. Pritchard, C. S. Bretherton, Interpreting and stabilizing machine-learning parametrizations of convection. *J. Atmos. Sci.* **77**, 4357–4375 (2020).

27. N. D. Brenowitz, C. S. Bretherton, Spatially extended tests of a neural network parametrization trained by coarse-graining. *J. Adv. Model. Earth Syst.* **11**, 2728–2744 (2019).

28. A. Kwa, S. K. Clark, B. Henn, N. D. Brenowitz, J. McGibbon, O. Watt-Meyer, W. A. Perkins, L. Harris, C. S. Bretherton, Machine-learned climate model corrections from a global storm-resolving model: Performance across the annual cycle. *J. Adv. Model. Earth Syst.* **15**, e2022MS003400 (2023).

29. Y. Han, G. J. Zhang, Y. Wang, An ensemble of neural networks for moist physics processes, its generalizability and stable integration. *J. Adv. Model. Earth Syst.* **15**, e2022MS003508 (2023).

30. J. Lin, S. Yu, L. Peng, T. Beucler, E. Wong-Toi, Z. Hu, P. Gentine, M. Geleta, M. Pritchard, Navigating the noise: Bringing clarity to ML parameterization design with O(100) ensembles. *J. Adv. Model. Earth Syst.* **17**, e2024MS004551 (2025).

31. D. Kochkov, J. Yuval, I. Langmore, P. Norgaard, J. Smith, G. Mooers, M. Klöwer, J. Lottes, S. Rasp, P. Düben, S. Hatfield, P. Battaglia, A. Sanchez-Gonzalez, M. Willson, M. P. Brenner, S. Hoyer, Neural general circulation models for weather and climate. *Nature* **632**, 1060–1066 (2024).

32. H. Hersbach, B. Bell, P. Berrisford, S. Hirahara, A. Horányi, J. Muñoz-Sabater, J. Nicolas, C. Peubey, R. Radu, D. Schepers, A. Simmons, C. Soci, S. Abdalla, X. Abellan, G. Balsamo, P. Bechtold, G. Biavati, J. Bidlot, M. Bonavita, G. de Chiara, P. Dahlgren, D. Dee, M.

Diamantakis, R. Dragani, J. Flemming, R. Forbes, M. Fuentes, A. Geer, L. Haimberger, S. Healy, R. J. Hogan, E. Hólm, M. Janisková, S. Keeley, P. Laloyaux, P. Lopez, C. Lupu, G. Radnoti, P. de Rosnay, I. Rozum, F. Vamborg, S. Villaume, J. N. Thépaut, The ERA5 global reanalysis. *Q. J. R. Meteorol. Soc.* **146**, 1999–2049 (2020).

33. D. A. Lavers, A. Simmons, F. Vamborg, M. J. Rodwell, An evaluation of ERA5 precipitation for climate monitoring. *Q. J. R. Meteorol. Soc.* **148**, 3152–3165 (2022).

34. G. Tang, M. P. Clark, S. M. Papalexiou, Z. Ma, Y. Hong, Have satellite precipitation products improved over last two decades? A comprehensive comparison of GPM IMERG with nine satellite and reanalysis datasets. *Remote Sens. Environ.* **240**, 111697 (2020).

35. W. E. Chapman, J. S. Schreck, Y. Sha, D. J. Gagne II, D. Kimpara, L. Zanna, K. J. Mayer, J. Berner, CAMulator: Fast emulation of the community atmosphere model. arXiv:2504.06007 (2025).

36. T. Gneiting, A. E. Raftery, Strictly proper scoring rules, prediction, and estimation. *J. Am. Stat. Assoc.* **102**, 359–378 (2007).

37. G. J. Huffman, D. T. Bolvin, D. Braithwaite, K.-L. Hsu, R. J. Joyce, C. Kidd, E. J. Nelkin, S. Sorooshian, E. F. Stocker, J. Tan, D. B. Wolff, P. Xie, "Integrated Multi-satellitE Retrievals for the Global Precipitation Measurement (GPM) mission (IMERG)" in *Satellite Precipitation Measurement: Volume 1* (Springer, 2020), pp. 343–353.

38. B. R. Johnston, W. J. Randel, J. P. Sjoberg, Evaluation of tropospheric moisture characteristics among COSMIC-2, ERA5 and MERRA-2 in the tropics and subtropics. *Remote Sens.* **13**, 880 (2021).

39. K. Krüger, A. Schäfler, M. Wirth, M. Weissmann, G. C. Craig, Vertical structure of the lower-stratospheric moist bias in the ERA5 reanalysis and its connection to mixing processes. *Atmos. Chem. Phys.* **22**, 15559–15577 (2022).

40. G. J. Huffman, R. F. Adler, A. Behrangi, D. T. Bolvin, E. J. Nelkin, G. Gu, M. R. Ehsani, The new version 3.2 Global Precipitation Climatology Project (GPCP) monthly and daily precipitation products. *J. Clim.* **36**, 7635–7655 (2023).

41. M. Nogueira, Inter-comparison of ERA-5, ERA-interim and GPCP rainfall over the last 40 years: Process-based analysis of systematic and random differences. *J. Hydrol.* **583**, 124632 (2020).

42. D. Watters, A. Battaglia, R. P. Allan, The diurnal cycle of precipitation according to multiple decades of global satellite observations, three CMIP6 models, and the ECMWF reanalysis. *J. Clim.* **34**, 5063–5080 (2021).

43. S.-H. Jiang, L.-Y. Wei, L.-L. Ren, L.-Q. Zhang, M.-H. Wang, H. Cui, Evaluation of IMERG, TMPA, ERA5, and CPC precipitation products over mainland China: Spatiotemporal patterns and extremes. *Water Sci. Eng.* **16**, 45–56 (2023).

44. Y. Xin, Y. Yang, X. Chen, X. Yue, Y. Liu, C. Yin, Evaluation of IMERG and ERA5 precipitation products over the Mongolian Plateau. *Sci. Rep.* **12**, 21776 (2022).

45. X. Wu, J. Su, W. Ren, H. Lü, F. Yuan, Statistical comparison and hydrological utility evaluation of ERA5-Land and IMERG precipitation products on the Tibetan Plateau. *J. Hydrol.* **620**, 129384 (2023).

46. P. Aryastana, P. I. Wahyuni, L. Dewi, J. P. Punay, I. G. N. H. R. Haditama, S. P. Jalakam, The quantitative comparison of grid re-analysis rainfall products, satellite rainfall products, and hourly rainfall gauge observation over Bali Province. *E3S Web Conf.* **445**, 01020 (2023).

47. Q. Sun, C. Miao, Q. Duan, H. Ashouri, S. Sorooshian, K.-L. Hsu, A review of global precipitation data sets: Data sources, estimation, and intercomparisons. *Rev. Geophys.* **56**, 79–107 (2018).

48. R. K. Pradhan, Y. Markonis, M. R. V. Godoy, A. Villalba-Pradas, K. M. Andreadis, E. I. Nikolopoulos, S. M. Papalexiou, A. Rahim, F. J. Tapiador, M. Hanel, Review of GPM IMERG performance: A global perspective. *Remote Sens. Environ.* **268**, 112754 (2022).

49. N. Herold, A. Behrangi, L. V. Alexander, Large uncertainties in observed daily precipitation extremes over land. *J. Geophys. Res. Atmos.* **122**, 668–681 (2017).

50. J. Zhang, K. Howard, C. Langston, B. Kaney, Y. Qi, L. Tang, H. Grams, Y. Wang, S. Cocks, S. Martinaitis, A. Arthur, K. Cooper, J. Brogden, D. Kitzmiller, Multi-radar multi-sensor (MRMS) quantitative precipitation estimation: Initial operating capabilities. *Bull. Am. Meteorol. Soc.* **97**, 621–638 (2016).

51. C. Guilloteau, E. Foufoula-Georgiou, "Multiscale evaluation of satellite precipitation products: Effective resolution of IMERG" in *Satellite Precipitation Measurement: Volume 2* (Springer, 2020), pp. 533–558.

52. Z. Zhou, D. Lu, B. Yong, Z. Shen, H. Wu, L. Yu, Evaluation of GPM-IMERG precipitation product at multiple spatial and sub-daily temporal scales over mainland China. *Remote Sens.* **15**, 1237 (2023).

53. S. Rasp, S. Hoyer, A. Merose, I. Langmore, P. Battaglia, T. Russell, A. Sanchez-Gonzalez, V. Yang, R. Carver, S. Agrawal, M. Chantry, Z. Ben Bouallegue, P. Dueben, C. Bromberg, J. Sisk, L. Barrington, A. Bell, F. Sha, WeatherBench 2: A benchmark for the next generation of data-driven global weather models. *J. Adv. Model. Earth Syst.* **16**, e2023MS004019 (2024).

54. I. Price, A. Sanchez-Gonzalez, F. Alet, T. R. Andersson, A. El-Kadi, D. Masters, T. Ewalds, J. Stott, S. Mohamed, P. Battaglia, R. Lam, M. Willson, GenCast: Diffusion-based ensemble forecasting for medium-range weather. arXiv:2312.15796 [cs.LG] (2023).

55. K.-Y. Cheng, L. Harris, C. Bretherton, T. M. Merlis, M. Bolot, L. Zhou, A. Kaltenbaugh, S. Clark, S. Fueglistaler, Impact of warmer sea surface temperature on the global pattern of intense convection: Insights from a global storm resolving model. *Geophys. Res. Lett.* **49**, e2022GL099796 (2022).

56. E. Hovmöller, The trough-and-ridge diagram. *Tellus* **1**, 62–66 (1949).

57. J. Norris, A. Hall, J. D. Neelin, C. W. Thackeray, D. Chen, Evaluation of the tail of the probability distribution of daily and subdaily precipitation in CMIP6 models. *J. Clim.* **34**, 2701–2721 (2021).

58. A. Dai, Global precipitation and thunderstorm frequencies. Part II: Diurnal variations. *J. Clim.* **14**, 1112–1128 (2001).

59. P. W. Battaglia, J. B. Hamrick, V. Bapst, A. Sanchez-Gonzalez, V. Zambaldi, M. Malinowski, A. Tacchetti, D. Raposo, A. Santoro, R. Faulkner, C. Gulcehre, F. Song, A. Ballard, J. Gilmer, G. Dahl, A. Vaswani, K. Allen, C. Nash, V. Langston, C. Dyer, N. Heess, D. Wierstra, P. Kohli, M. Botvinick, O. Vinyals, Y. Li, R. Pascanu, Relational inductive biases, deep learning, and graph networks. arXiv:1806.01261 [cs.LG] (2018).

60. V. Eyring, S. Bony, G. A. Meehl, C. A. Senior, B. Stevens, R. J. Stouffer, K. E. Taylor, Overview of the coupled model intercomparison project phase 6 (CMIP6) experimental design and organization. *Geosci. Model Dev.* **9**, 1937–1958 (2016).

61. M. Wheeler, G. N. Kiladis, Convectively coupled equatorial waves: Analysis of clouds and temperature in the wavenumber–frequency domain. *J. Atmos. Sci.* **56**, 374–399 (1999).

62. B. Madieros, Wavenumber-frequency (2023); https://github.com/brianpm/wavenumber_frequency.

63. C. Barnet, Sounder SIPS: AQUA AIRS IR-only Level 3 CLIMCAPS: Comprehensive Quality Control Gridded Daily V2 (Goddard Earth Sciences Data and Information Services Center, 2019); https://disc.gsfc.nasa.gov/datasets/SNDRAQIL3CDCCP-2/summary.

64. V. V. Kharin, F. W. Zwiers, X. Zhang, M. Wehner, Changes in temperature and precipitation extremes in the CMIP5 ensemble. *Clim. Change* **119**, 345–357 (2013).

65. S. Pfahl, P. A. O'Gorman, E. M. Fischer, Understanding the regional pattern of projected future changes in extreme precipitation. *Nat. Clim. Chang.* **7**, 423–427 (2017).

66. B. Asadieh, N. Y. Krakauer, Global trends in extreme precipitation: Climate models versus observations. *Hydrol. Earth Syst. Sci.* **19**, 877–891 (2015).

67. P. A. O'Gorman, Precipitation extremes under climate change. *Curr. Clim. Change Rep.* **1**, 49–59 (2015).

68. G. A. Young, Alternative smoothed bootstraps. *J. R. Stat. Soc. Series B Stat. Methodol.* **52**, 477–484 (1990).

69. S. Westra, L. V. Alexander, F. W. Zwiers, Global increasing trends in annual maximum daily precipitation. *J. Clim.* **26**, 3904–3918 (2013).

70. J. Lu, L. Ruby Leung, Q. Yang, G. Chen, W. D. Collins, F. Li, Z. Jason Hou, X. Feng, The robust dynamical contribution to precipitation extremes in idealized warming simulations across model resolutions. *Geophys. Res. Lett.* **41**, 2971–2978 (2014).

71. H. Tabari, Climate change impact on flood and extreme precipitation increases with water availability. *Sci. Rep.* **10**, 13768 (2020).
